# Two-Step Ligand-Directed Covalent Fluorescent Labeling of the Adenosine A_1_‑Receptor That Maintains Its Orthosteric Binding Site’s Availability to Bind Ligands

**DOI:** 10.1021/acs.jmedchem.5c02389

**Published:** 2026-01-08

**Authors:** Chia-Yang Lin, Simon Platt, Joelle Goulding, Stephen J. Briddon, Nicholas D. Kindon, Clare R. Harwood, Chih-Wei Lai, Barrie Kellam, Stephen J. Hill

**Affiliations:** † School of Pharmacy, Division of Bimolecular Science and Medicinal Chemistry, Biodiscovery Institute, 6123 University of Nottingham, Nottingham NG7 2RD, U.K.; ‡ Centre of Membrane Proteins and Receptors (COMPARE), University of Birmingham and University of Nottingham, The Midlands, Nottingham NG7 2UH, U.K.; § School of Life Sciences, Division of Physiology, Pharmacology and Neuroscience, University of Nottingham, Nottingham NG7 2UH, U.K.; ∥ School of Pharmacy, National Defense Medical University, Taipei 114201, Taiwan

## Abstract

Genetic tagging of G protein-coupled receptors (GPCRs) with bioluminescent or fluorescent proteins is a well-established method for the study of ligand-binding and protein–protein interactions using resonance energy transfer approaches. Here we present a two-step, ligand-directed covalent labeling (LDCL) method that allows attachment of different fluorescent labels to an untagged adenosine A_1_ receptor using click chemistry. We also describe a range of biophysical approaches to confirm that the orthosteric binding site remains available to interact with endogenous ligands, agonists and antagonists, and access to the orthosteric binding site is not sterically hindered by the transferred cargo (fluorophore or click-reactive group).

## Graphical abstract

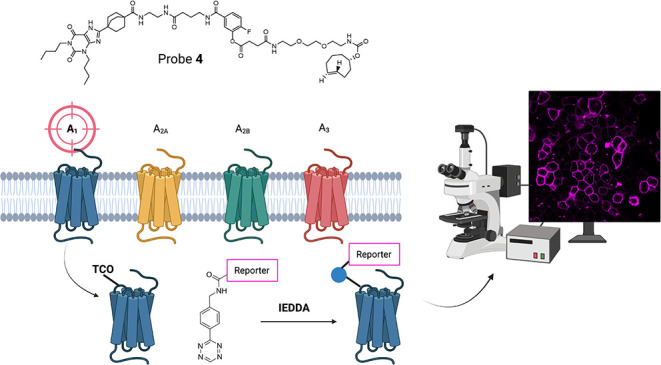

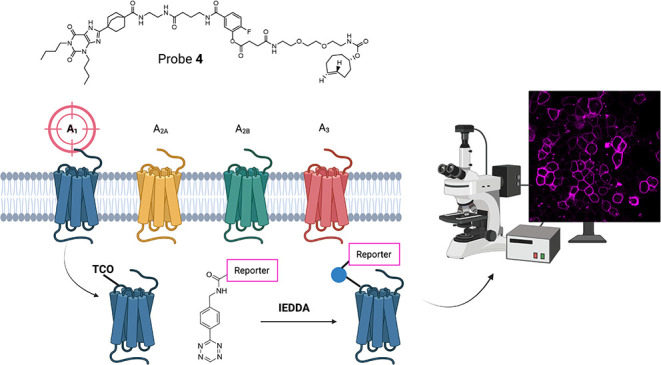

## Introduction

The ability to monitor ligand–receptor and protein–protein interactions has been revolutionized by genetically engineered GPCRs tagged with bioluminescent or fluorescent proteins and the application of bioluminescence resonance energy transfer (BRET), Förster resonance energy transfer (FRET) and single particle tracking approaches.^1–4^ However, these approaches require expression of genetically modified GPCRs. Fluorescent ligands offer a powerful alternative approach to investigate ligand–receptor interactions at endogenous receptor levels in native cellular environments,^5–9^ but their reversible nature can hamper evaluation of both temporal and spatial aspects of GPCR signaling.

The adenosine A_1_-receptor (A_1_AR) is one of the four GPCRs that are activated by the endogenous purine nucleoside adenosine.^10,11^ The A_1_AR has modulatory roles in the cardiovascular, respiratory and renal systems^12–15^ as well as a neuromodulatory role in astrocyte signaling in the central nervous system.^16–18^ Recent studies have also shown that targeting the A_1_AR with allosteric modulators may provide novel therapeutic strategies for the treatment of neuropathic pain.^19^ To shed further light on the role of A_1_ARs in health and disease, we and others have developed reversible fluorescent ligands (both agonists and antagonists) that target the A_1_AR.^3,7,20,21^

Ligand-directed covalent labeling (LDCL) is an alternative strategy whereby an orthosteric ligand for the GPCR of interest is conjugated to a reporter moiety via an electrophilic reactive linker that can react with a nucleophilic amino acid side chain (e.g., lysine) of the receptor and covalently transfer the chemical cargo (e.g., fluorescent reporter).^22–25^ The utility of this approach, however, relies heavily on the receptor-selectivity of the intact conjugate and the ability of the guiding orthosteric ligand to be rapidly released from its binding site following covalent cargo transfer. It is also important that the transferred cargo (fluorophore or click-reactive group) does not sterically hinder access to the orthosteric site by endogenous ligands and other drugs.

Here we report on the development of a two-step labeling strategy using a polyethylene glycol (PEG) linked reactive strained alkene, *trans*-cyclooctene (TCO), group as the covalently transferred cargo to selectively label the A_1_AR. The TCO group is a highly strained dienophile that undergoes an ultrafast inverse electron demand Diels–Alder (IEDDA) click reaction with tetrazines.^24,26,27^ Probe **4** (Figure 1a) was synthesized by modifying the linker and substituent positions of the previously reported ligand (probe **5**) by Comeo et al. (2024).^24^ The structural design of probe **4** was inspired by analogs of A_1_AR LDCL probes (see Supporting Information, Table 1). These analogs share the same orthosteric ligand and cargo—sulfoCy5—but differ in linker composition (glycine or γ-aminobutyric acid) and in the positioning of fluorine and ester substituents on the phenyl ring. Notably, the combination of γ-aminobutyric acid and a *meta*-fluoro-phenyl ester in compound **S5** resulted in more than a 2-fold increase in A_1_/A_2A_ selectivity compared to compound **S1** (Comeo et al. 2024 reported),^24^ which incorporated β-alanine and a *para*-fluoro-phenyl ester. Additionally, **S5** exhibited the highest Bmax in a NanoBRET-based A_1_AR saturation binding assay, suggesting improved cargo transfer efficiency.

**Figure 1:**
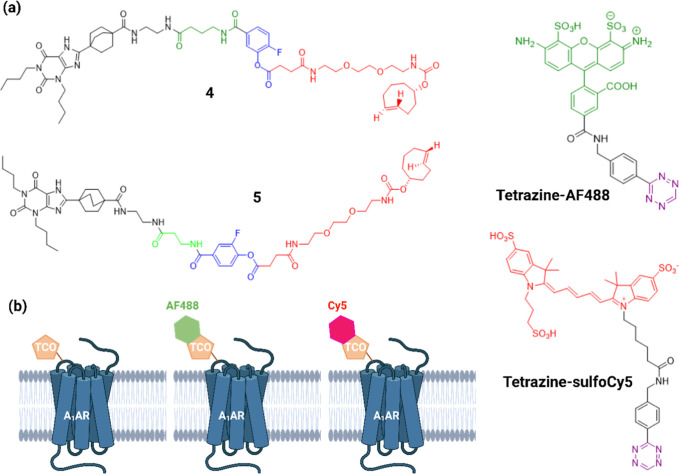
Probes for labeling of the A_1_ AR. (a) Structures of probe **4** derived from the Comeo et al. reported ligand-directed covalent probe **5**,^24^ the click-reactive red (Cy5) and green (AF488) tetrazine derivatives. (b) Schematic showing how red and green fluorophores can be attached to the covalently transferred *trans*-cyclooctene (TCO) group. Created in BioRender. Lai, C. (2026) https://BioRender.com/ets30wv.

**Table 1:** Affinities of Probe **4** ^a^

p*K*_*i*_				
ligand\AR	NL-hA_1_	NL-hA_2A_	NL-hA_2B_	NL-hA_3_
Probe **4**	7.78 ± 0.13 (5)	5.73 ± 0.05 (4)	5.39 ± 0.10 (5)	<5 (5)

a Apparent p*K*_i_ values were calculated for probe **4** using NanoBRET competition binding assays with 15 nM CA200645 as the fluorescent ligand. All data are presented as the mean ± SEM from *n* independent experiments (indicated in parentheses). NL-hA_1_, NL-hA_2B_, and NL-hA_3_ ARs were stably expressed in HEK293 cells while NL-hA_2A_ ARs were transiently expressed in HEK293T cells.

Docking simulations were conducted using probe **4** and the A_1_AR crystal structure (PDB ID: 5UEN) via Discovery Studio Client (Figure 2). The results indicated that probe **4** not only retains strong binding affinity for A_1_AR but also positions its reactive moiety closer to lysine 168 (3.6 Å), compared to probe **5** (6.6 Å), which was previously assumed to label this residue.^24^ This configuration is proposed to preserve orthosteric binding accessibility for subsequent ligand engagement while facilitating covalent transfer of the polyethylene glycol (PEG)-TCO group to lysine residues (K168 or K173) located near the binding pocket. The TCO group covalently transferred by probe **4** can then rapidly react with a range of tetrazine-conjugated fluorophores (Figure 1a,b). We have also used novel biophysical approaches that confirm that the orthosteric binding site remains available to interact with endogenous ligands, agonists and antagonists following LDCL labeling of the A_1_AR with a fluorophore.

**Figure 2:**
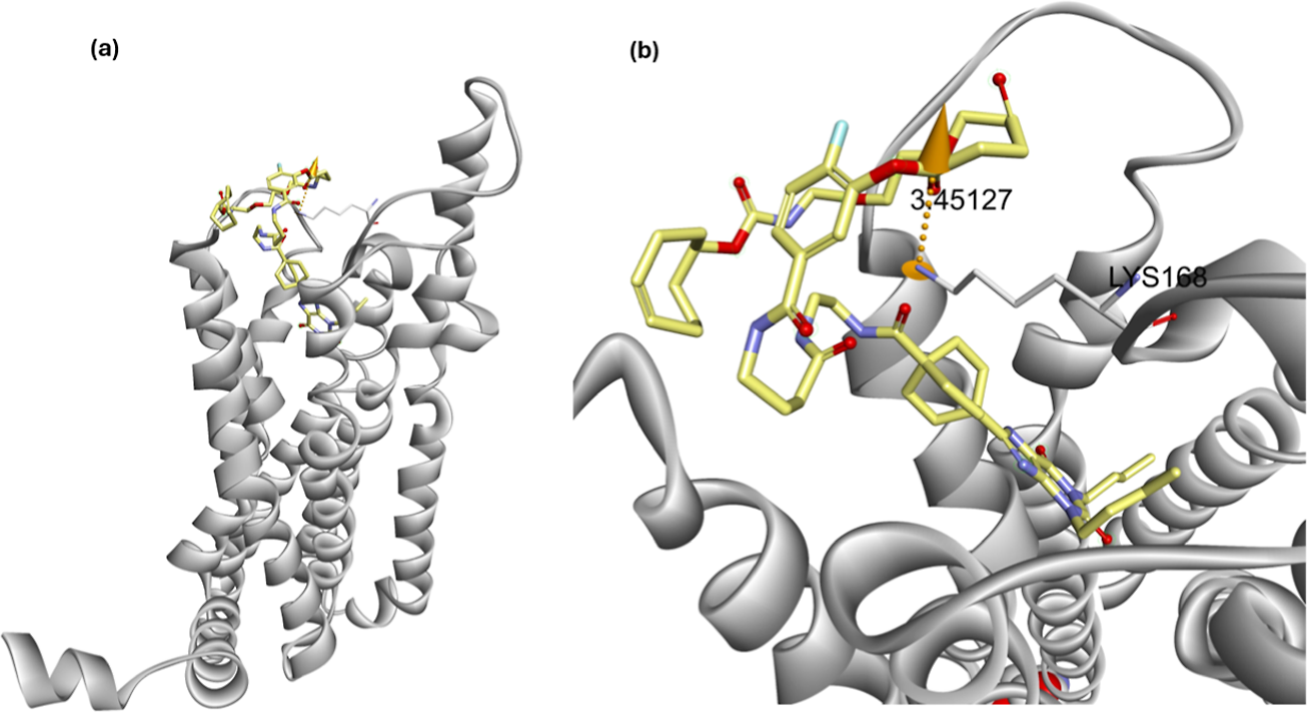
Docking simulation of probe **4** with the Human A_1_ adenosine receptor (hA1AR, PDB ID:5UEN). The hA_1_AR is shown as gray ribbon structures, and probe **4** is depicted with a yellow (licorice) backbone. Docking simulations were performed using Discovery Studio Client, with both the receptor and ligand subjected to structure preparation and energy minimization prior to docking via the CDOCKER algorithm.^28^ (a) displays the overall receptor architecture with probe **4** (shown in yellow licorice) bound at the orthosteric site. (b) Provides a magnified view of the binding pocket entrance, highlighting Lys168 as white sticks. The predicted distance between the amine group of Lys168 and the electrophilic carbonyl carbon of the phenyl ester moiety in probe **4** is 3.45 Å, which is shorter than the 6.6 Å observed for probe **5** in a previous study by Comeo et al. (2024).^24^ These simulation results suggest that modifying probe **5**′s linker from β-alanine to γ-aminobutyric acid and shifting the ester attachment position from *para* to *meta* may preserve A_1_AR binding affinity while potentially enhancing cargo transfer efficiency.

## Results

### Chemistry

The LDCL probe facilitates cargo transfer to the target receptor via a nucleophilic substitution reaction between its electrophilic moiety and nucleophilic side chains of amino acid residues of the receptor.^29^ The spatial proximity and orientation of these reactants are critical for efficient labeling. When appropriately aligned, the intermolecular reaction mimics a pseudointramolecular process, enhancing reaction efficiency.^29,30^ To optimize these parameters, we substituted β-alanine with γ-aminobutyric acid and repositioned the ester group from the *para*-to the *meta*-position based on the docking simulation (Figure 2), resulting in the modified probe **4**. The synthetic route for probe **4** is illustrated in Scheme 1.

**Scheme 1:**
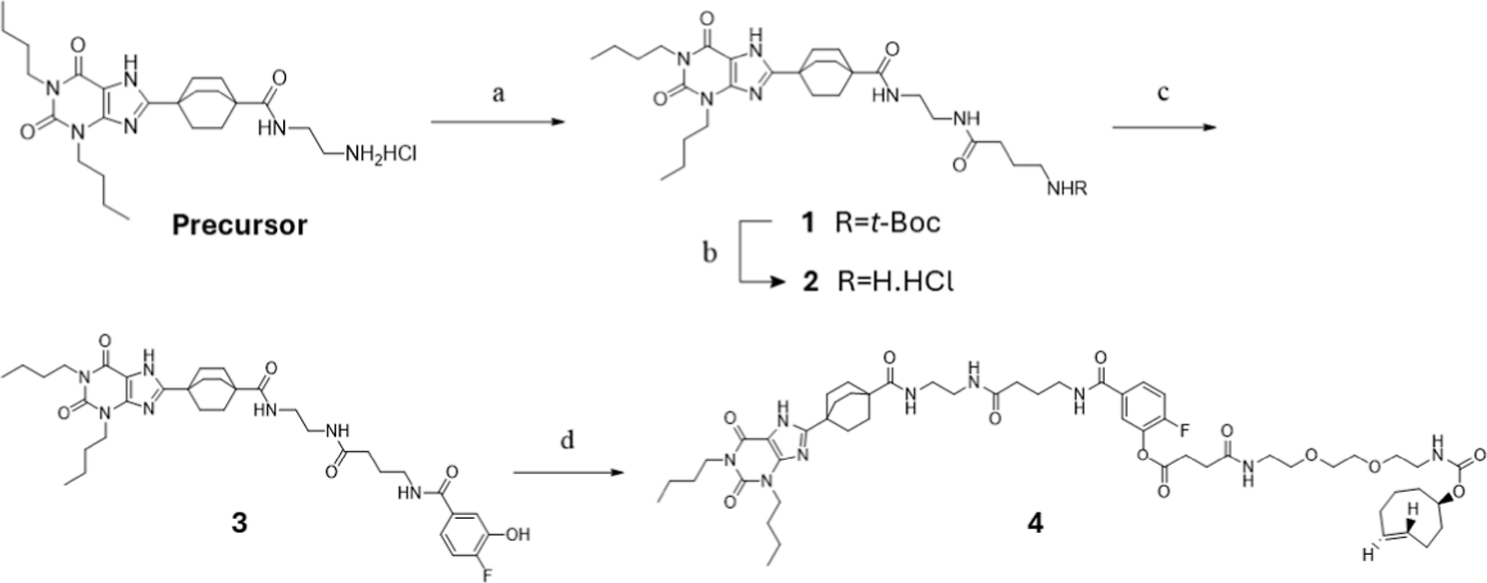
Synthesis of LDCL Probe **4** ^a^ ^24^

a Reagents and conditions: (a) boc-γ-aminobutyric acid, COMU, DIPEA, rt, 2 h, 78%; (b) 4 N HCl in dioxane, rt, 1 h; (c) 4-fluoro-3-hydroxybenzoic acid, COMU, DIPEA, DMF, 90 °C, overnight, 49%; (d) PEG-linked TCO carboxylic acid, BEP, DIPEA, DMF, rt, avoid light, overnight, 49%.

The preparation of the hydrochloride salt precursor was previously described by Comeo et al. (2024).^24^ The synthesis began with amide coupling of Boc-γ-aminobutyric acid and the precursor using COMU ([(1-cyano-2-ethoxy-2-oxoethylidenaminooxy)­dimethylamino-morpholino-carbenium hexafluorophosphate]) and DIPEA, yielding compound **1**. Subsequent acidolytic Boc deprotection afforded the corresponding hydrochloride salt (compound **2**), which was then coupled with 4-fluoro-3-hydroxybenzoic acid using COMU and DIPEA. Heating the reaction overnight at 90 °C minimized ester formation and favored production of the phenol derivative (compound **3**) as the major product. A TCO-tethered carboxylic acid, preactivated with 2-bromo-1-ethylpyridinium tetrafluoroborate (BEP) and DIPEA, was then coupled with compound **3** to yield the final product (probe **4**). Probe **4** was characterized by analytical reversed-phase high-performance liquid chromatography (RP-HPLC), confirming purity above 97%. High-resolution mass spectrometry revealed a molecular weight within 10 ppm of the calculated value. Structural confirmation was further supported by ^1^H and ^13^C NMR spectroscopy.

## Pharmacology

### LDCL of the Human A_1_AR

We assessed the apparent affinity of probe **4** for the A_1_AR using competition NanoBRET-based ligand binding studies with the reversible fluorescent adenosine receptor antagonist CA200645 (Figure 3a,b; Table 1). Probe **4** had an apparent p*K*_*i*_ of 7.78 for the human A_1_AR and exhibited >100-fold selectivity over the other three human adenosine receptor subtypes (Figure 3b; Table 1). Covalent transfer of the PEG-TCO group to the A_1_AR was confirmed using HEK293G cells stably expressing Twin-Strep-SNAP-A_1_AR. This construct features an A_1_AR engineered with a Twin-Strep tag and a SNAP tag at the N-terminus. The Twin-Strep tag binds specifically to Strep-Tactin, a high-affinity streptavidin variant, and offers enhanced binding affinity compared to a single Strep tag, while retaining reversible binding through biotin competition.^31^ This design facilitates efficient purification of A_1_AR from cell lysates. The SNAP tag enables covalent labeling with its substrate (e.g., SNAP-tag substrate AF647),^32^ serving as a positive control. Cells were labeled for 1 h with 200 nM of probe **4**, followed by a 1 h incubation with 500 nM tetrazine-sulfoCy5. After labeling, cells were solubilized and A_1_AR was purified using MagStrep magnetic beads. SDS-PAGE gel electrophoresis and in-gel fluorescence confirmed that sulfoCy5 had been covalently transferred to the A_1_AR (Figure 4). Pretreatment of cells with 10 μM DPCPX completely prevented labeling of the A_1_AR by probe **4** (Figure 4).

**Figure 3:**
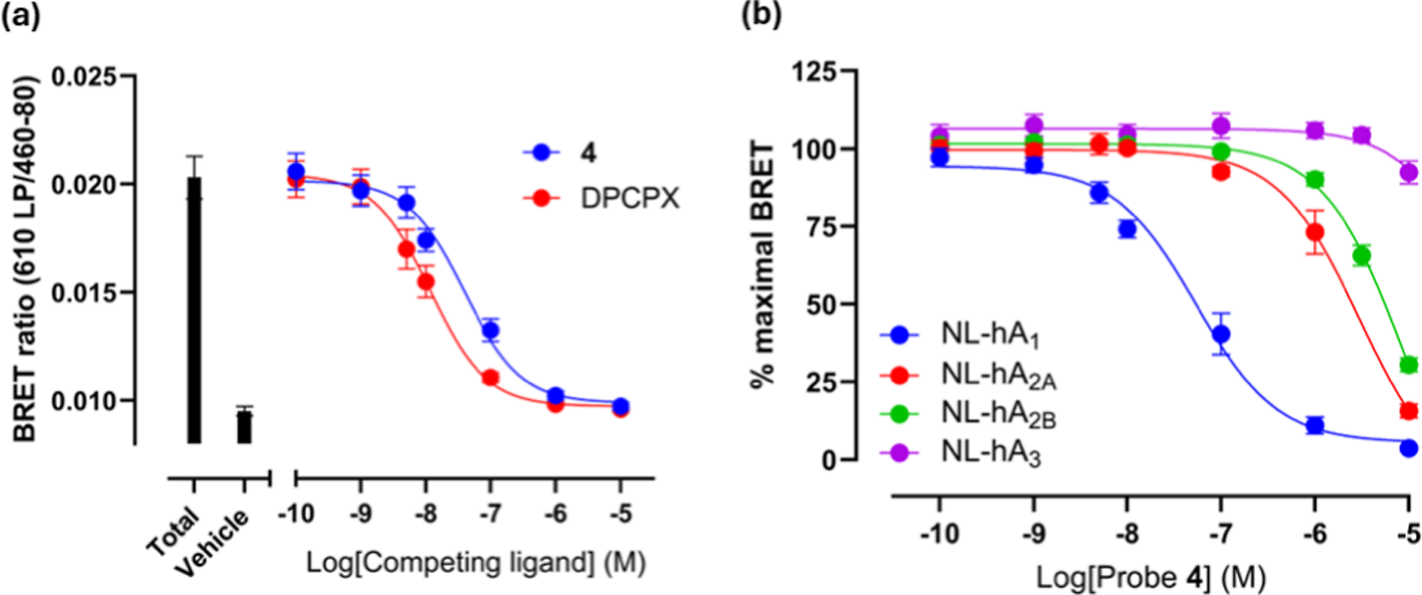
Binding of probe **4** to NLuc-adenosine receptors. (a) BRET signal for NL-hA_1_AR treated with 15 nM CA200645 or vehicle control and increasing concentrations of Probe **4** or DPCPX. (b) Specific binding BRET signal for NL-hA_1_, NL-hA_2A_, NL-hA_2B_ or NL-hA_3_ ARs treated with 15 nM CA200645 and increasing concentrations of probe **4**. Nonspecific binding for NL-hA_1_, NL-hA_2A_, NL-A_2B_ or NL-hA_3_ ARs was determined with 10 μM DPCPX, 10 μM ZM241385, 10 μM PSB603 or 10 μM MRS1220 respectively. Data represent the mean ± SEM from 4 (NL-hA_2A_) or 5 (other adenosine receptors) independent experiments conducted in triplicate.

**Figure 4:**
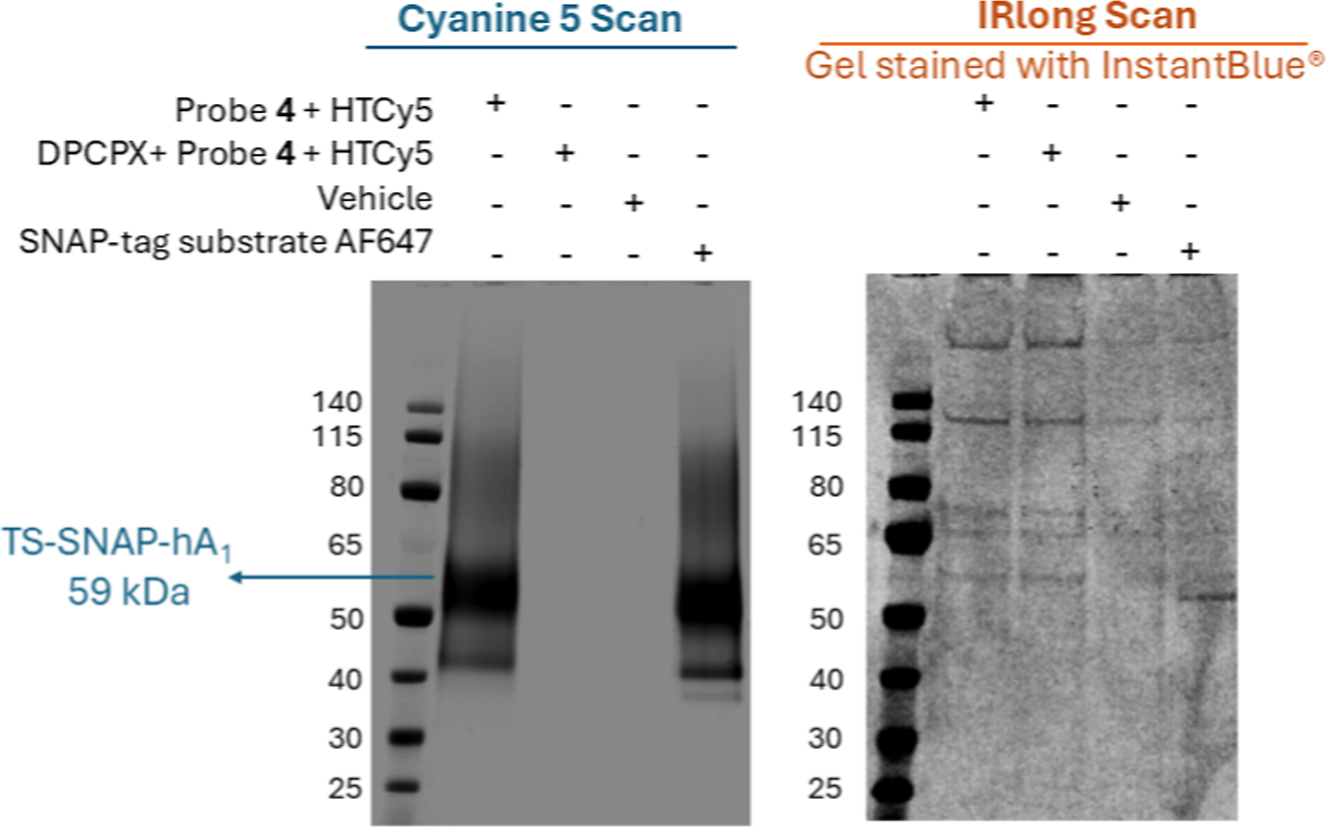
Covalent labeling of TS-SNAP-hA_1_ARs. HEK293G cells expressing TS-SNAP-A_1_AR were labeled with 200 nM of probe **4** for 1 h in the presence or absence of 10 μM DPCPX followed by incubation with 500 nM Tetrazine-sulfoCy5 (HTCy5) for a further hour. Where DPCPX was used, cells were preincubated for 30 min with DPCPX before addition of probe **4**. Vehicle (DMEM without phenol red) and SNAP-tag substrate AF647 served as negative and positive controls, respectively. In the Cy5 scan (left panel), a fluorescent band was present in cells without DPCPX pretreatment and absent in cells pretreated with DPCPX. The band position was consistent with the positive control and matched the calculated protein weight (59 kDa) for the TS-SNAP-hA_1_AR. The InstantBlue stained gel image (right panel) confirmed that protein samples were loaded into the gel. The first lane of each gel shows the PageRuler prestained protein ladder with the molecular weight in kDa. All the incubations were conducted at 37 °C and the gel-scanning was done at room temperature. Images are representative of three independent experiments.

To assess the time course of the click reaction for attachment of fluorophores to the LDLC transferred PEG-TCO group on the A_1_AR, we compared the efficiency of the click reaction for two concentrations of tetrazine-sulfoCy5 and methyl-tetrazine-sulfoCy5 using NanoBRET in cells expressing N-terminal Nluc-A_1_AR following LDCL with probe **4** (Figure 5). These studies showed that attachment of sulfoCy5 was very much slower with methyl-tetrazine-sulfoCy5 (Figure 5b) than with tetrazine-sulfoCy5 (Figure 5a).^33^ In the case of tetrazine-sulfoCy5, full labeling could be achieved rapidly in a concentration-dependent manner (Figure 5a), indicating that short labeling periods in intact cells should keep nonspecific labeling to a minimum.

**Figure 5:**
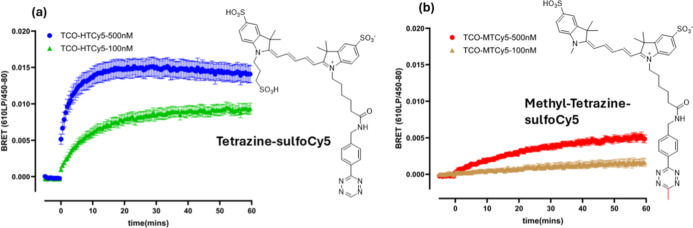
Time course of click chemistry attachment of tetrazine-sulfoCy5 or methyl-tetrazine-sulfoCy5 to the LDCL transferred TCO group. Cells expressing NL-hA_1_AR were LDCL tagged with 200 nM probe **4** to transfer PEG-TCO to the A_1_AR. Following furimazine addition, BRET ratios were recorded for 5 min (at 30 s intervals) before addition of 100 or 500 nM tetrazine-sulfoCy5 (a) or 100 or 500 nM methyl-tetrazine-sulfoCy5 (b) at time zero. BRET measurements were then made every 30 s for a further 60 min. All the incubation and measurement were conducted at 37 °C. Values represent the mean ± SEM of six replicates from five independent experiments.

In HEK 293T cells transiently transfected with SNAP-hA_1_AR and treated with probe **4**, a short 15 min incubation with 1 μM tetrazine-SulfoCy5 (HTCy5) was sufficient to tag cell surface A_1_ARs with Cy5 (Figure 6a,b). Pretreatment of SNAP-hA_1_AR cells with 10 μM DPCPX, however, significantly reduced cell surface labeling with Cy5 (Figure 6a,b), and revealed a low level of nonspecific binding (Figure 6a). In contrast, if the SNAP-hA_1_ARs were labeled with SNAP-tag substrate AF488, there was no reduction of the cell surface signal by DPCPX pretreatment. There was, however, a small significant increase in cell surface AF488-tagged A_1_ARs which is most likely attributed to prevention of internalization of these receptors by endogenous adenosine (Figure 6b).

**Figure 6:**
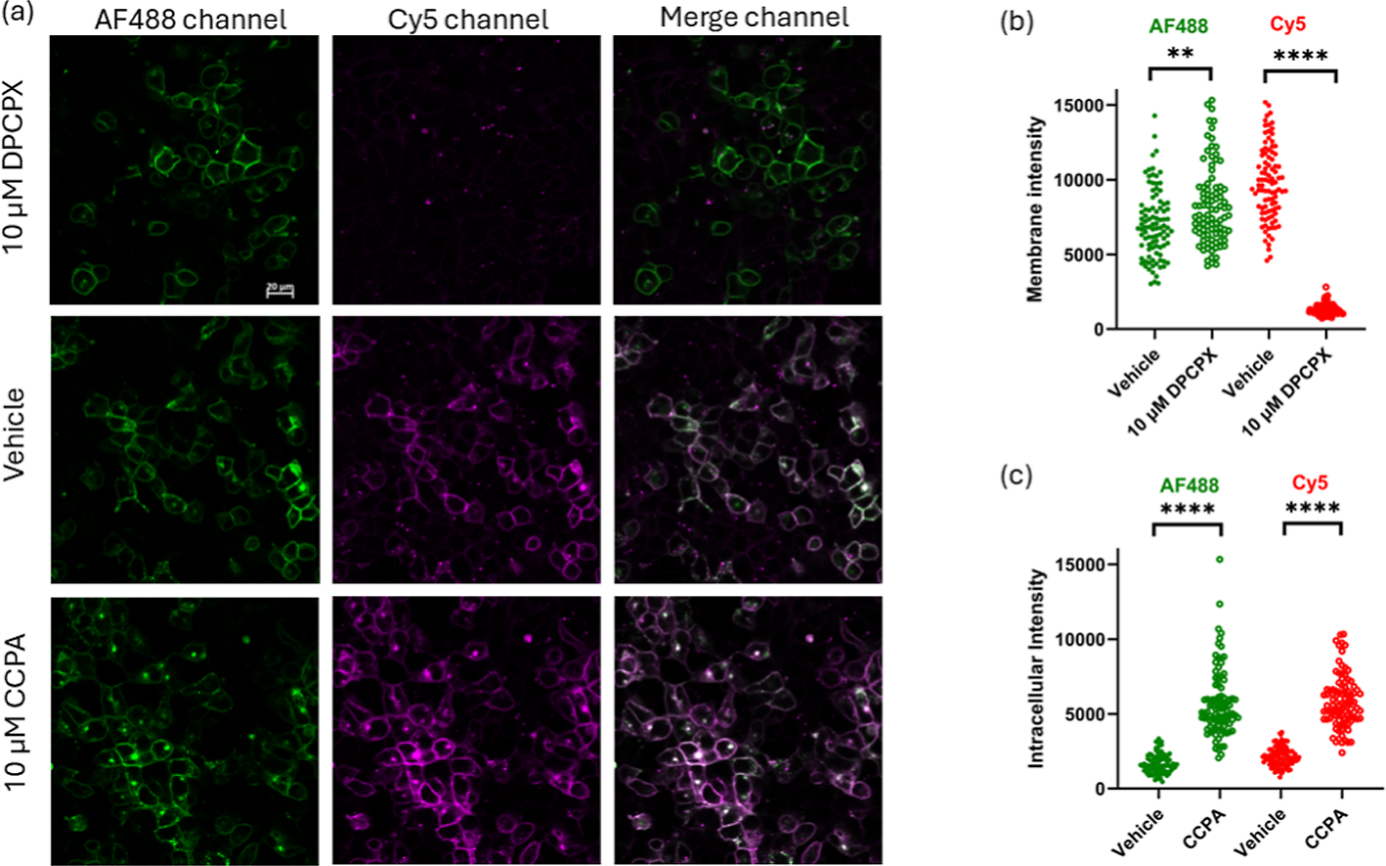
Confocal imaging of an LDCL-transferred fluorophore attached to the A_1_AR. (a) HEK293T cells transiently expressing SNAP-hA_1_ARs were labeled with 250 nM SNAP-tag substrate AF488. Subsequently, cells were incubated with probe **4** (100 nM) for 2 h (in the absence or presence of 10 μM DPCPX) at 37 °C and then incubated with HTCy5 (1 μM) for 15 min prior to imaging. Cells were then incubated for 2 h in the absence (upper and middle frames) or presence (bottom frames) of 10 μM CCPA at 37 °C. Scale bar represents 20 μm. Images are representative of six independent experiments conducted in duplicate. (b,c) Intensity measurement for AF488 and Cy5 channels for regions of interest drawn using ImageJ for (b) membrane or (c) intracellular regions. Data are individual values for 96 cells measured for each condition. (b) **P < 0.01 for the increase in AF488 signal in the presence of DPCPX. ****p < 0.0001 for the decrease in the Cy5 signal with DPCPX. (c) ****P < 0.0001 for the increase in the presence of CCPA relative to the corresponding vehicle control.

### Availability of the Orthosteric Binding Site of the A_1_AR Following LDCL

An essential requirement of this LDCL approach is that the orthosteric element used for direct binding and subsequent covalent labeling can rapidly leave the orthosteric binding site, and access to the orthosteric site by the endogenous ligand or other drugs is not restricted by steric hindrance from the transferred cargos. As a first step to demonstrate this, we treated SNAP-A_1_AR cells previously labeled with probe **4** and tetrazine-sulfoCy5, with the A_1_AR selective agonist 2-chloro-*N* ^6^-cyclopentyladenosine (CCPA, 10 μM) for 2 h (Figure 6a lower panels, Figure 6c). This led to significant internalization of the Cy5-labeled A_1_ARs which paralleled the internalization of SNAP-tag-AF488 labeled receptors (Figure 6a,c). We also used NanoBRET to examine whether a reversible red fluorescent ligand (probe **6**; Supplementary Figure 2) could access the orthosteric site of A_1_ARs pretreated with probe **4** and tagged with tetrazine-AF488 (Figure 7a–f). Interestingly, the BRET signal for the binding of probe **6** was enhanced in the red channel due to FRET between AF488 and the BODIPY630/650 of probe **6** (Figure 7a,b,f; Supplementary Figure 3). This was paralleled by a concentration dependent decrease in BRET (Figure 7d) in the green channel due to FRET-based energy loss. The binding affinities determined for probe **6** from these data in the red and green channels (in the presence or absence of the probe **4** AF488 tag) were not significantly different (Table 2). Fluorescence lifetime imaging microscopy (FLIM)^34^ and FRET imaging confirmed the significant decrease in donor lifetime of A_1_ARs prelabeled with probe **4** and tetrazine-AF488 (Figure 8) because of FRET between AF488 and the BODIPY630/650 of probe **6** (Figure 8c).

**Figure 7:**
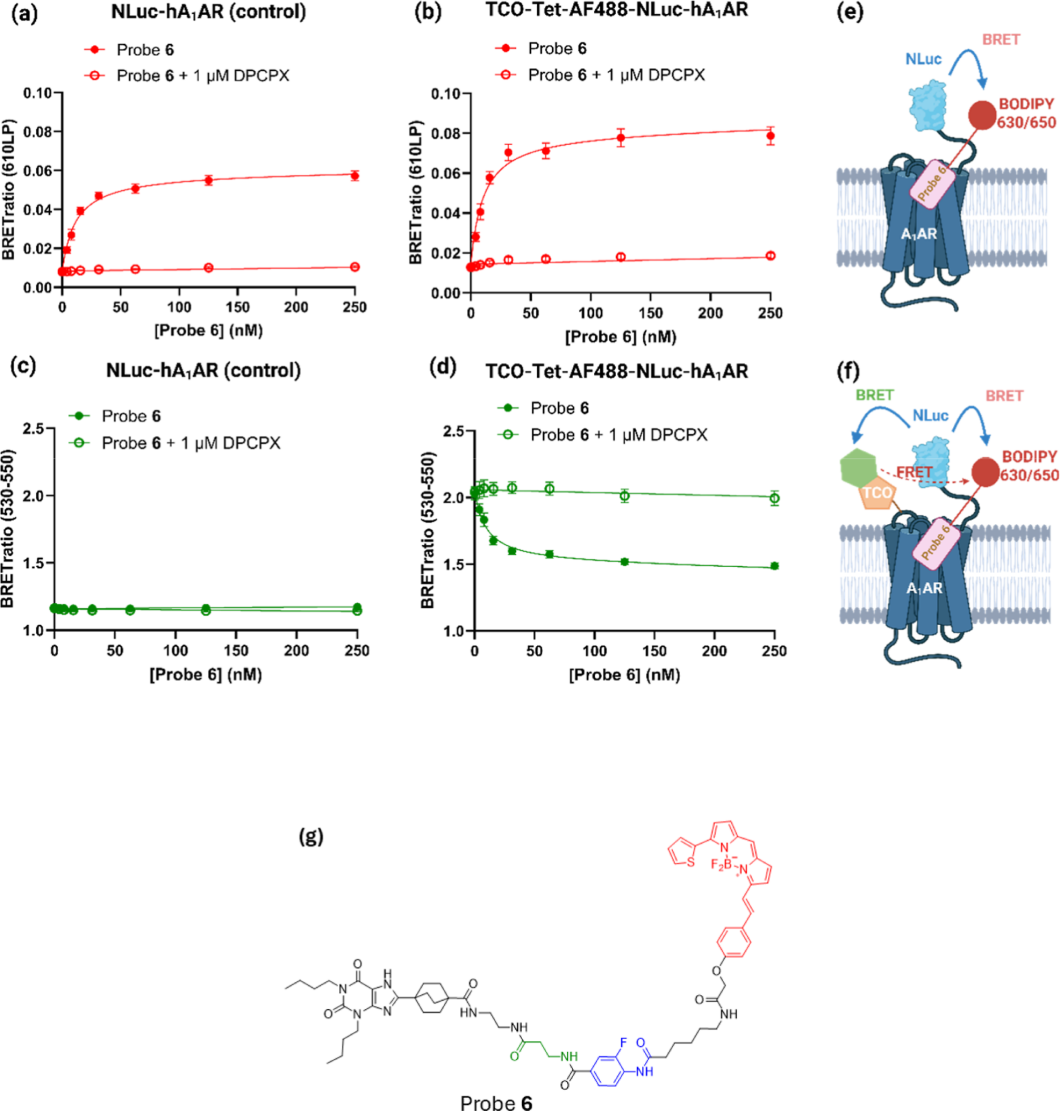
Availability of the A_1_AR orthosteric binding site to bind the reversible red fluorescent probe **6** following LDCL with probe **4** and click-attachment of a green AF488 fluorophore. (a,b) BRET signals (measured in the red Cy5 channel) for binding of increasing concentrations of the reversible probe **6** to (a) cells expressing NL-hA_1_ARs or (b) cells expressing NL-hA_1_ARs which have been tagged with TCO-Tet-AF488. (c,d) Probe **6** binding measured in the green channel for cells expressing (c) NL-hA_1_ARs or (d) NL-hA_1_ARs which have been tagged with TCO-Tet-AF488. Nonspecific binding was determined in cells preincubated with 1 μM DPCPX for 30 min. All incubation steps and BRET measurements were conducted at 37 °C. All data represent the mean ± SEM from six independent experiments conducted in triplicate. (e,f) Schematics showing BRET between the nanoluciferase on the N terminus of the receptor and the bound fluorescent probe **6**. In (f) additional BRET can occur between NLuc and the green fluorophore which can lead to FRET between green and red fluorophores and an enhanced red signal. (g) The structure of probe **6**. Created in BioRender. Lai, C. (2026) https://BioRender.com/jiae0uc.

**Table 2:** *K*_D_ Values for Probe **6** Determined from NanoBRET Saturation Assays in the Presence or Absence of LDCL-Transferred TCO and Tetrazine-AF488 Labeling^a^

NL-hA_1_AR	*K*_D_ of probe **6** (*n*)
control/red channel	12.27 ± 2.03 nM (6)
+ TCO-Tetrazine-AF488/red channel	10.92 ± 1.88 nM (6)
+ TCO-Tetrazine-AF488/green channel	9.73 ± 1.66 nM (6)

a Measurements were made in cells expressing NL-hA_1_ARs or cells expressing NL-hA_1_ARs which have been tagged with AF488 via incubation with probe **4** and tetrazine-AF488. BRET measurements were made in the red or green channels. For red BRET measurements, emissions were read at 450 nm (80 nm bandpass; donor NanoLuc emission) and >610 nm (long pass; fluorescent probe **6** emission). For parallel green BRET measurements, emissions were read at 475 nm (30 nm bandpass; donor NanoLuc emission) and at 535 nm (30 nm bandpass; AF488 tag emission). Values represent mean ± SEM from *n* independent experiments (indicated in parentheses).

**Figure 8:**
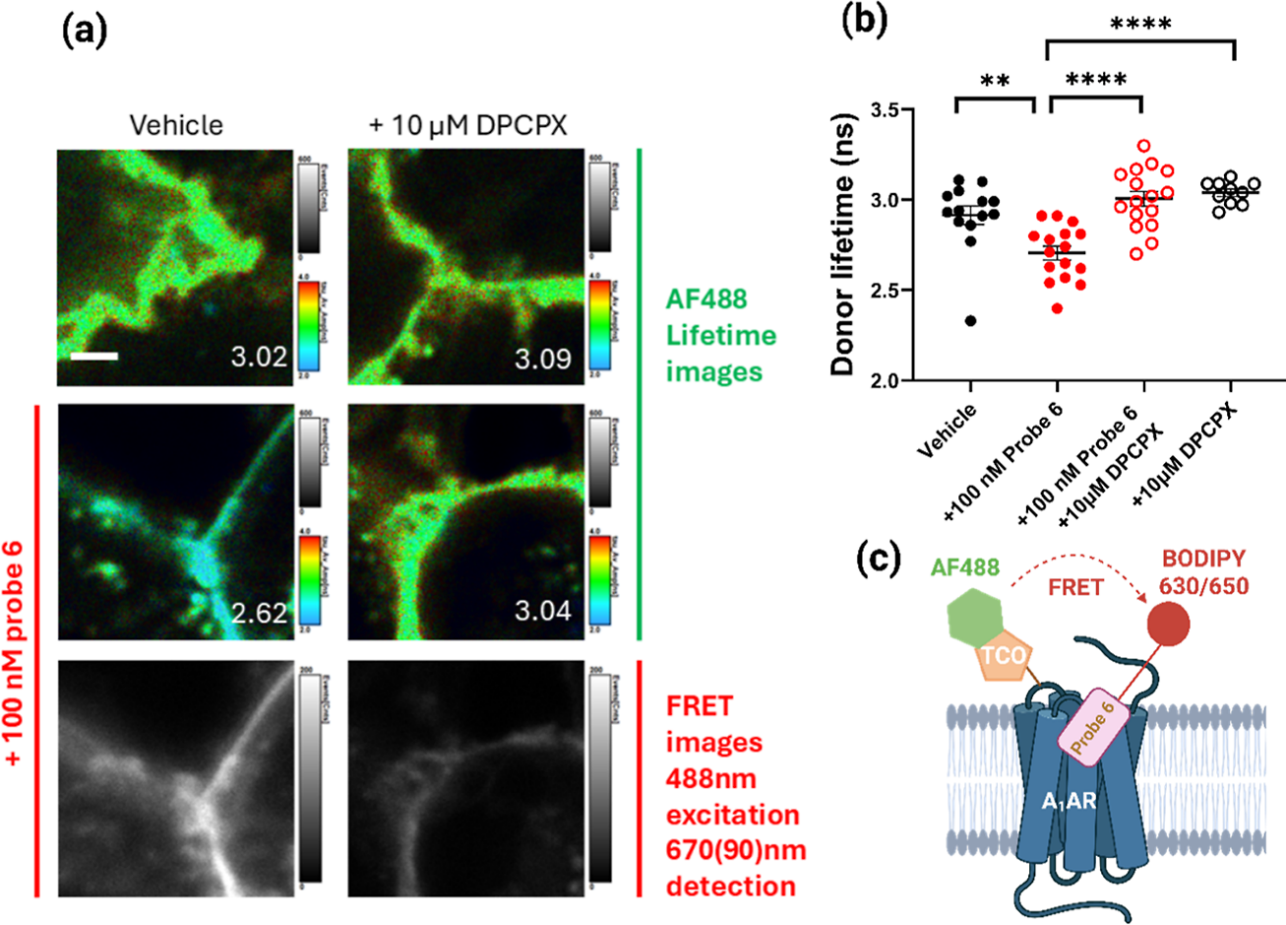
FLIM-FRET imaging of cells following LDCL with probe **6** and subsequent click chemistry with tetrazine-AF488. (a) FLIM-FRET images following binding of probe **6** to A_1_ARs that have previously been covalently labeled via LDCL with probe **4** and tetrazine-AF488. Upper four panels show representative donor lifetime images and determined lifetimes (ns). Images are pseudocolored for average amplitude-weighted lifetime. Scale bar represents 2 μm. Lower 2 image panels show acceptor signal as a result of FRET after 488 nm excitation, following 100 nM probe **6** treatment in the absence or presence of 10 μM DPCPX. (b) Plotted single-cell donor lifetime measurements, *n* = 10–16 over 3–5 experimental dates. Mean and SEM are also shown. **p < 0.01 or ****p < 0.0001 compared to probe **6** alone (one-way ANOVA with Tukey multiple comparison test). (c) Schematic showing FRET between the TCO-attached AF488 and the BODIPY 630/650 of probe **6**. Created in BioRender. Lai, C. (2026) https://BioRender.com/qlz2ys0.

To further confirm that individual receptor species can be initially labeled with a single green fluorophore (tetrazine-AF488) and subsequently bind with a red fluorescent (probe **6**) ligand, fluorescence cross-correlation spectroscopy (FCCS) experiments were performed.^8,35,36^ This technique was used to determine whether the red and green fluorophore-tagged species were diffusing together within the cell membrane. Co-focused laser beams were used to create overlapping confocal detection volumes which encompass *circa* 0.3 μm of the cell membrane and where both fluorescent species could be excited. The fluorescence emissions from the red and green channels were collected independently using two separate detectors. To minimize spectral crosstalk between channels,^35,36^ we used pulsed interleaved excitation to provide a phase delay so that each photon arriving at the detector could be assigned to a specific excitation pulse. The fluorescence fluctuations collected for both red and green channels were then cross-correlated to determine the presence of fluorescence intensity peaks that were simultaneously detected in both channels. This analysis allowed the number (75.10 ± 8.52 N/μm^2^) of codiffusing particles (red and green) within the confocal volume to be determined (Figure 9).

**Figure 9:**
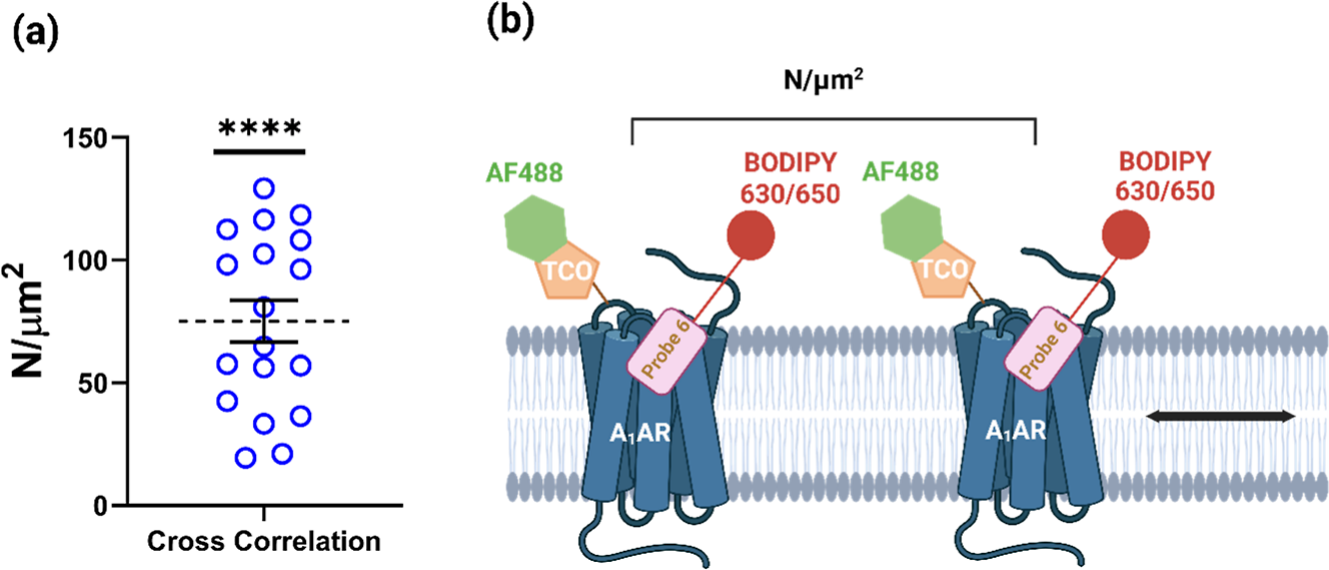
Pulsed interleaved excitation fluorescence cross correlation spectroscopy (FCCS) of cells following LDCL with probe **4** and subsequent click chemistry with tetrazine-AF488. (a) FCCS following binding of probe **6** to A_1_ARs that have previously been covalently labeled via LDCL using probe **4** and tetrazine-AF488. The *y* axis shows the particle numbers (N/μm^2^) of cross-correlated red and green diffusing species within the confocal volume on the cell membrane, following treatment of AF-488 labeled cells with 100 nM probe **6**. Values show individual particle numbers obtained on 18 cells in five separate experiments. The mean and SEM are also shown. ****p < 0.0001 compared to zero particle number (Wilcoxon signed rank test). (b) Schematic illustrating that particle number represents receptors diffusing in the membrane that are dual labeled with TCO-AF488 and probe **6**. Created in BioRender. Lai, C. (2026) https://BioRender.com/zimgzcm.

## Discussion and Conclusions

This study has demonstrated that two-step LDCL provides a powerful approach to tag cell surface GPCRs without the need for genetic modification of the receptor. The transfer of a PEG-TCO reactive group to the A_1_AR using LDCL has allowed the subsequent attachment of two different-colored fluorophores using tetrazine-sulfoCy5 or tetrazine-AF488 to achieve a rapid click chemistry mediated transfer of cargo in intact living cells. Appropriate choice of tetrazine-conjugated fluorophore concentration and incubation time allowed rapid subsequent fluorescent labeling of cells and maintained nonspecific fluorophore attachment at extremely low levels. In this respect, the use of tetrazine-conjugated fluorophores was far superior to the methyl-tetrazine-conjugates that we have used previously.^24^ Using short incubation times with tetrazine-sulfoCy5, it was notable, that there was negligible attachment of Cy5 fluorophores to the cell membrane in cells that had been pretreated with the selective A_1_AR antagonist DPCPX prior to incubation with the LDCL probe **4**. This is in marked contrast to the high nonspecific binding detected with a different GPCR using a modular click ligand-directed approach.^25^ Covalent transfer of a fluorophore to the A_1_AR was confirmed by labeling the receptor prior to detergent solubilization, receptor purification and SDS-PAGE gel electrophoresis. Subsequent in gel fluorescence confirmed the presence of a fluorescently tagged A_1_AR with the expected molecular weight of 59 kDa. Again, as with our imaging studies, pretreatment of cells with DPCPX before LDCL with probe **4** completely prevented the fluorescent labeling of the purified A_1_AR.

The utility of this LDCL approach, however, relies heavily on the receptor-selectivity of the intact conjugate and the ability of the guiding orthosteric ligand to be rapidly released from its binding site following covalent cargo transfer. It is also important that the transferred cargo (fluorophore or click-reactive group) does not sterically hinder access to the orthosteric site by endogenous ligands and other drugs. Selectivity of probe **4** was confirmed by evaluating its ability to inhibit the binding of the nonselective fluorescent adenosine receptor ligand CA200645 ^3^ to the hA_1_AR with high apparent affinity and its much weaker (*circa* 100-fold lower affinity) binding to hA_2A_, hA_2B_ and hA_3_ receptors.

Key features of the present study, however, are the biophysical approaches that have been utilized to confirm that the orthosteric binding site remains available to bind ligands after LDCL labeling of the A_1_AR and subsequent click chemistry attachment of a fluorophore. Confocal imaging studies confirmed that the tagged A_1_AR could be internalized following treatment with CCPA in keeping with previous observations of A_1_AR internalization.^37^ However, to demonstrate that the orthosteric binding site of the A_1_AR was still available to bind ligands after LDCL tagging with tetrazine-AF488 we examined whether AF488-tagged A_1_ARs could still bind a reversible red fluorescent A_1_AR ligand. The reversible ligand (probe **6**) that we chose to do this was very similar in structure to probe **5** ^24^ but contained a BODIPY 630/650 fluorophore in place of the PEG-TCO group, as well as a 2-fluorophenylamide group (instead of a 2-fluorophenyl ester of probe **5**) which does not covalently transfer cargo to the A_1_AR receptor. We confirmed that its binding could be rapidly reversed by addition of DPCPX (Supplementary Figure 2). Binding of probe **6** to AF488-tagged NLuc-A_1_ARs was monitored using NanoBRET^3^ and it was notable that following LDCL tagging of the A_1_AR with tetrazine-AF488, the NanoBRET signal in the red detection channel was enhanced by FRET between the AF488 of the A_1_AR and the BODIPY 630/650 fluorophore on probe **6**. Evaluation of the NanoBRET signal in the green BRET channel was consistent with energy transfer occurring from the AF488 to BODIPY630/650 resulting in a concentration-dependent reduction in BRET ratio in the green channel as the binding of probe **6** to the A_1_AR increased. The proximity requirements of NanoBRET (<10 nm)^3,38^ therefore confirm that the orthosteric binding site was available to bind ligands after LDCL tagging with tetrazine-AF488 and that the A_1_AR bound ligands with similar affinity to untagged receptors (Table 2).

Th energy transfer between the covalently attached AF488 of the A_1_AR, following LDCL tagging of the A_1_AR with tetrazine-AF488, and the BODIPY 630/650 fluorophore on probe **6** was also confirmed using fluorescence lifetime imaging of the AF488 channel where a significant drop in donor lifetime was noted on binding of probe **6** to the A_1_AR which could be prevented by addition of the selective A_1_ antagonist DPCPX. FRET between AF488 and BODIPY 630/650 was also directly demonstrated (Figure 8), again confirming that probe **6** could readily bind to LDCL-tagged A_1_ARs. However, the NanoBRET, fluorescence lifetime and FRET studies all rely on resonance energy transfer to confirm the close proximity of probe **6** and LDCL-tagged A_1_ARs. As a consequence, we also investigated a technique that was independent of resonance energy transfer.

Fluorescence cross correlation spectroscopy (FCCS) with pulsed interleaved excitation^8,35,36^ was used to determine whether the red fluorophore of probe **6** would diffuse together with the LDCL-transferred green tag of the AF488-A_1_ARs in the same fluorescent particles within the cell membrane. The technique uses cofocused laser beams to create overlapping confocal detection volumes (0.25–0.5 femtolitres) which encompass *circa* 0.3 μm^2^ of the cell membrane and where both fluorescent species can be excited. The fluorescence emissions from the red and green channels were collected independently and then cross-correlated to determine the presence of fluorescence intensity peaks that were simultaneously detected in both channels. This analysis revealed that a significant number (*p* < 0.0001; 75.10 ± 8.52 N/μm^2^) of codiffusing particles (red and green) were detected within the confocal volume. These data, taken together, strongly suggest that probe **6** is bound to AF488-labeled A_1_ARs and indicate that the orthosteric site of the A_1_AR is available to bind ligands and is not sterically hindered by the transferred fluorescent cargo.

In summary, we have optimized a two-step ligand-directed covalent labeling ligand and demonstrated this ligand then allows attachment of different fluorescent labels to an untagged A_1_AR using click chemistry. We have also used a range of biophysical approaches (confocal imaging, NanoBRET, FRET, lifetime imaging, FCCS) to confirm that the orthosteric binding site is available to interact with endogenous ligands, agonists and antagonists, and that access to the orthosteric binding site is not sterically hindered by the transferred cargo (fluorophore or click-reactive group).

## Experimental Section

### Chemistry

Chemicals and solvents (analytic and HPLC grade) were acquired from commercial suppliers without further purification. *trans*-Cyclooctene-NHS (TCO-NHS) ester was obtained from Jena Biosciences (Germany). Methyl-tetrazine-sulfoCy5 was purchased from Lumiprobe (Germany), and tetrazine-sulfoCy5 was acquired from BroadPharm (USA). Thin-layer chromatography (TLC) was used to monitor reaction status, and TLC plates were commercial products (Merk Kieselgel 60 F). Visualization of TLC was under UV light at 254 nm, followed by a ninhydrin stain. Purification via automated flash column chromatography was carried out with an Interchim PuriFlash 4100 system (PF4100–250) coupled to a dual-wavelength DAD UV detector (200–600 nm) using silica high performance (HP) 50 μm cartridges. Methods were designed and exerted via Interchim Flash (ver: V5.1c.09) software. Compound was purified at a flow rate of 20 mL/min with a gradient program with 98–90% solvent B over 20 min (solvent A: DCM, solvent B: MeOH). Reverse-phase high performance liquid chromatography (RP-HPLC) was conducted with Waters 515 LC system compact with a Waters 996 photodiode array detector at wavelength between 190 to 800 nm. Spectra were analyzed via Millennium 32 software. Compound purified through RP-HPLC was accompanied by semipreparative YMC-Pack C8 column (150 mm × 10 mm × 5 μm) at a flow rate of 4 mL/min using a gradient method from 30 to 95% solvent B over 16 min (solvent A = 0.1% formic acid in H_2_O, solvent B = 0.1% formic acid in CH_3_CN). Compound purity analysis was performed with YMC-Pack C8 analytic column (150 mm × 4.6 mm × 5 μm) at a flow rate of 1 mL/min with a gradient method from 5 to 95% solvent B over 20 min (solvent A = 0.1% formic acid in H_2_O, solvent B = 0.1% formic acid in MeCN). Final products presented a single peak in RP-HPLC accompanied photodiode array spectra and were >95% pure. NMR spectra were acquired through a Bruker-AV 400. ^1^H NMR spectra were recorded at 400.13 MHz, and ^13^C NMR was recorded at 101.62 MHz. All ^13^C NMR are ^1^H broadband decoupled. Deuterium solvents applied in NMR analysis (reference peak listed) were CDCl_3_: (δ H = 7.26 ppm, δ C = 77.16 ppm) purchased from Cambridge Isotope Laboratories Inc., CD_3_OD: (δ H = 3.34 ppm, δ C = 49.86 ppm), and DMSO-*d*_6_: (δ H = 2.5 ppm, δ C = 40.45 ppm) supplied by Sigma-Aldrich (UK). Chemical shifts (δ) are recorded in part per million (ppm), and coupling constants are recorded in Hz. Signal split patterns are depicted as the following abbreviation: singlet (s), doublet (d), triplet (t), quadruplet (q), pentet (p), broad (br), doublet of doublets (dd), double doublet of doublets (ddd), double triplet of doublets (dtd) and multiplet (m). Software Mnova 14.2.2 was used to analyze NMR data. Preliminary low-resolution mass spectra (LRMS) data were acquired through the Shimadzu UFLCXR LC–MS system coupled with an Applied Biosystems API2000 and visualized at 254 nm (channel 1) and 220 nm (channel 2). LC–MS was performed at a flow rate of 0.5 mL/min over a 5 min period with a Phenomenex Gemini-NX C18 110A column (50 mm × 2 mm × 3 μm). Running buffers were as follows: buffer A, 0.1% formic acid in H_2_O; buffer B, 0.1% formic acid in MeCN. Method A analyzed samples with a gradient method of solvent B from 5 to 95 and back to 5% across 5 min. High-resolution mass spectra (HRMS) were acquired through a Bruker microTOF mass spectrometer by electrospray ionization operating in negative ion mode.

### Molecular Docking

Docking simulations between Probe **4** and the human A_1_ adenosine receptor (hA_1_AR, PDB ID: 5UEN) were performed using Discovery Studio Client v24.1.023298. Prior to docking, both the receptor and ligand structures underwent preparation and energy minimization using the CHARMM force field.^28^ Protein preparation was performed virtually with the pH parameter set to physiological conditions (pH: 7.4), while potential ligand structures—including isomers, tautomers, and various protonation states—were considered across a pH range of 6.5 to 8.5. Probe **4** structures generated under the docking pH condition (7.5 ± 1.0) were individually docked into the processed hA_1_AR using the CDOCKER algorithm.^28^ The docking region was defined as a spherical volume with a 20 Å radius centered on the orthosteric binding pocket. From the top ten docking poses, the most stable ligand–receptor complex was selected for further analysis. The distance between the amine group of Lys168 and the electrophilic carbonyl carbon of the phenyl ester moiety in probe **4** was measured to evaluate proximity relevant to potential covalent cargo transfer.

### General Procedure a COMU Facilitated Amide Coupling

A solution of the respective carboxylic acid (1.0 equiv) in anhydrous DMF (2 mL) was stirred with DIPEA (1.1 equiv) and COMU (1.1 equiv) for 5 min. Then, a solution of the respective amine (1 equiv) in anhydrous DMF (3 mL) was added to the activated carboxylic acid mixture, and the reaction was stirred for 30–60 min. LC–MS was used to monitor the progress of the reaction. Upon completion, iced water (50 mL) was added to the mixture. If the product precipitated, it was collected by filtration. If no precipitation occurred, the mixture was extracted with EtOAc. The collected EtOAc extract was washed sequentially with 1 M HCl_(aq)_, saturated NaHCO_3(aq)_, and brine. The washed EtOAc solution was dried over anhydrous MgSO_4_, filtered, and evaporated under reduced pressure. Finally, the resulting residue was purified using automated flash column chromatography.

### General Procedure B Phenyl Ester Synthesis

A solution of the respective carboxylic acid (1.0 equiv) in anhydrous DMF (0.5 mL) was stirred with DIPEA (2 drops) and 2-bromo-1-ethyl-pyridinium tetrafluoroborate (BEP) (1 equiv) for 5 min. Then, a solution of the respective phenol (1 equiv) in anhydrous DMF (0.5 mL) was added to the activated carboxylic acid mixture, and the reaction was left overnight in the dark. LC–MS was used to monitor the reaction progress. Upon completion, DMF was removed under reduced pressure. The residue was reconstituted with 0.5 mL of MeCN and 1 mL of H_2_O. The target compound was purified and collected via semipreparative RP-HPLC. The collected fraction was concentrated and lyophilized to afford a desired product.

### General Procedure C *t*-Boc Deprotection

*t*-Boc-protected amine was dissolved in 4 M HCl/dioxane (4 mL, 0.5 mL for 1 mg scale reaction) and stirred from 20 to 60 min at rt. The reaction was monitored by TLC and LC-MS. The acidic solvent was removed under vacuum within a fume hood. The HCl salt of the desired amine was obtained and used in the next step without further purification.

#### Preparation of *tert*-Butyl (4-((2-(4-(1,3-dibutyl-2,6-dioxo-2,3,6,7-tetrahydro-1*H*-purin-8-yl)­bicyclo­[2.2.2]­octane-1-carboxamido)­ethyl)­amino)-4-oxobutyl)­carbamate (**1**)

Crude Precursor (247.5 mg, 0.50 mmol, 1 equiv) was reacted with Boc-γ-aminobutyric acid (120 mg, 0.59 mmol, 1.2 equiv). Reaction steps followed General Procedure A. Reaction was monitored by LC–MS. The workup method was described in General procedure A. The red crude product was rinsed with DCM and a light-yellow precipitate formed. The yellow precipitate was collected via gravity filtration and dried in an oven overnight to give **1** (247.8 mg, 0.38 mmol, yield = 78%). LC-MS *m*/*z* calcd for C_33_H_53_N_7_O_6_ [M – H^+^] 644.41; found 644.4, *t*_R_ = 2.95 min, Method A. ^1^H NMR (DMSO-*d*_6_): δ 12.9 (s, 1H), 7.79 (t, *J* = 5.6 Hz, 1H), 7.42 (t, *J* = 5.5 Hz, 1H), 6.79 (t, *J* = 5.24 Hz, 1H), 3.96 (t, *J* = 7.2 Hz, 2H), 3.86 (t, *J* = 7.4 Hz, 2H), 3.08 (t, *J* = 2.6 Hz, 4H), 2.89 (q, *J* = 7 Hz, 2H), 2.03 (t, *J* = 7.5 Hz, 2H), 1.88–1.84 (m, 6H), 1.74–1.70 (m, 6H), 1.61 (m, *J* = 7.3, 1.65 Hz, 4H), 1.5 (p, *J* = 7.8 Hz, 2H), 1.36 (s, 9H), 1.27 (h, *J* = 7.5 Hz, 4H), 0.90 (t, *J* = 7.44 Hz, 3H), 0.88 (t, *J* = 7.24 Hz, 3H). ^13^C NMR (CD_3_OD): δ 180.5, 176.1, 162.2, 158.6, 155.9, 152.8, 149.4, 108.2, 79.9, 44.2, 42.1, 40.9, 40.7, 40.1 39.9, 34.9, 34.3, 31.23, 31.21, 31.0, 29.2, 28.8, 27.3, 21.6, 20.8, 14.18, 14.12.

#### Preparation of *N*-(2-(4-Aminobutanamido)­ethyl)-4-(1,3-Dibutyl-2,6-Dioxo-2,3,6,7-tetrahydro-1*H*-purin-8-yl)­bicyclo­[2.2.2]­octane-1-carboxamide Hydrochloride Salt (**2**)

**1** (100 mg, 0.155 mmol, 1 equiv) was performed with General Procedure C. LC–MS was used to monitor the reaction. Once the *t*-Boc group was removed, the mixture was evaporated to afford the crude product **2** for the next step without further purification.

#### Preparation of 4-(1,3-Dibutyl-2,6-dioxo-2,3,6,7-tetrahydro-1*H*-purin-8-yl)-*N*-(2-(4-(4-fluoro-3-hydroxybenzamido)­butanamido)­ethyl)­bicyclo­[2.2.2]­octane-1-carboxamide (**3**)

Crude product **2** obtained from **1** (100 mg, 0.155 mmol, 1 equiv) *t*-boc deprotection was reacted with 4-fluoro-3-hydroxybenzoic acid (24.5 mg, 0.156 mmol, 1.01 equiv) and followed General Procedure A. The reaction was heated to 90 °C and stirred overnight. The reaction was monitored by LC–MS. The workup method was as no precipitate formed when water was poured into the mixture. Purification was by automated flash column (gradient MeOH: DCM from 5:95 to 10:90, 19CV) and afforded **3** (51.5 mg, 0.076 mmol, yield = 49%). LC-MS *m*/*z* calcd for C_35_H_48_FN_7_O_6_ [M – H^+^] 682.37; found 682.2, *t*_R_ = 2.75 min, Method A. ^1^H NMR (CD_3_OD): δ 7.43 (dd, *J* = 8.44, 2.1 Hz, 1H), 7.30 (ddd, *J* = 8.5, 4.22, 2.26 Hz, 1H), 7.12 (dd, *J* = 10.76, 8.56 Hz, 1H), 4.1 (t, *J* = 7.28 Hz, 2H), 3.99 (t, *J* = 7.5 Hz, 2H), 3.41 (t, *J* = 6.9 Hz, 2H), 3.31 (s, 4H), 2.00–1.86 (m, 14H), 1.73 (p, *J* = 7.42 Hz, 2H), 1.62 (p, *J* = 7.54 Hz, 2H), 1.38 (h, *J* = 7.2 Hz, 4H), 0.98 (t, *J* = 7.32 Hz, 3H), 0.97 (t, *J* = 7.36 Hz, 3H). ^13^C NMR (CD_3_OD): δ 180.5, 176.1, 169.4, 162.13, 156.0, 155.0 (d, *J* = 244.5 Hz), 152.8, 149.4, 146.4 (d, *J* = 13.27 Hz), 132.4, 119.9 (d, *J* = 7.21 Hz), 118.3 (d, *J* = 3.79 Hz), 116.8 (d, *J* = 19.33 Hz), 108.2, 44.2, 42.1, 40.7, 40.4, 40.1, 40.0, 34.9, 34.5, 31.23, 31.20, 30.98, 29.2, 26.7, 21.2, 20.8, 14.18, 14.11.

#### Preparation of 5-((4-((2-(4-(1,3-Dibutyl-2,6-dioxo-2,3,6,7-tetrahydro-1*H*-purin-8-yl)­bicyclo­[2.2.2]­octane-1-carboxamido)­ethyl)­amino)-4-oxobutyl)­carbamoyl)-2-fluorophenyl (*R*,*E*)-1-(cyclooct-4-en-1-yloxy)-1,12-dioxo-5,8-dioxa-2,11-diazapentadecan-15-oate (**4**)

**3** (1.71 mg, 2.51 μmol, 1.07 equiv) and (*R*,*E*)-1-(cyclooct-4-en-1-yloxy)-1,12-dioxo-5,8-dioxa-2,11-diazapentadecan-15-oic acid (0.94 mg, 2.35 μmol, 1 equiv) were reacted followed General procedure B. The crude product was purified through RP-HPLC with YMC C8 semipreparative column and the collected fraction was concentrated followed with lyophilization to give a white fluffy solid (1.24 mg, 1.16 μmol, yield = 49%). ^1^H NMR (400 MHz, CDCl_3_-*d*_3_): δ 11.26 (s, 1H), 8.07 (s, 1H), 7.76–7.70 (m, 1H), 7.67 (d, *J* = 7.1 Hz, 1H), 7.31 (s, 1H), 7.21 (t, *J* = 9.0 Hz, 1H), 6.89 (s, 1H), 6.67 (s, 1H), 6.46 (s, 1H), 5.60–5.51 (m, 1H), 5.51–5.42 (m, 1H), 5.14 (s, 1H), 4.33 (s, 1H), 4.09 (t, *J* = 7.4 Hz, 2H), 4.00 (t, *J* = 7.6 Hz, 2H), 3.71–3.15 (m, 18H), 2.97 (s, 2H), 2.67 (t, *J* = 6.6 Hz, 2H), 2.41–2.25 (m, 5H), 1.92 (dd, *J* = 10.8, 4.9 Hz, 13H), 1.86–1.52 (m, 17H), 1.45–1.29 (m, 5H), 0.96 (t, *J* = 7.32 Hz, 3 Hz), 0.93 (t, *J* = 7.32 Hz, 3H). ^13^C NMR (101 MHz, CDCl_3_-*d*_3_): δ 178.70, 174.21, 170.93, 168.61, 166.21, 163.16, 156.43 (d, *J* = 213.5 Hz), 151.33, 148.74, 138.14, 135.05, 133.11, 126.47, 123.36, 116.98 (d, *J* = 20.2 Hz), 106.83, 77.36, 43.52, 41.29, 39.94, 39.02, 38.81, 34.41, 34.06, 33.72, 32.67, 31.11, 30.36, 30.29, 30.04, 29.28, 28.41, 25.22, 20.40, 20.07, 14.01, 13.92. HRMS (TOF ESI^+^) calcd for C_54_H_78_FN_9_O_12_ [M + H]^+^: 1064.582674, found 1064.5834, error within 2.4 ppm. RP-HPLC with YMC analytic C8 column showed probe **4** retention as 19.476 min over 30 min analysis, purity = 97%.

## Pharmacology-General Method

### Reagents

Dulbecco’s Modified Eagle’s Medium (DMEM) with phenol red (D6546), DMEM without phenol red (D1145), and phosphate-buffered saline (PBS) were purchased from Sigma Chemicals (Pool, Dorset, UK). Fetal calf serum (FCS) was acquired from PAA Laboratories (Teddington, Middlesex, UK). Geneticin (G418) was obtained from Life Technologies (Paisley, UK). Optimem, MOPS SDS Running Buffer (20X), NUPAGE LDS sample buffer (4X), PageRuler Prestained Protein Ladder, NuPAGE 4–12% Bis-Tris gel (1.0 mm X 10 well), and Laury maltose neopentyl glycol (LMNG) were purchased from Thermo Fisher Scientific (USA). SNAP-surface Alexa Fluor 488 and 647 were acquired from New England Biolabs (Hitchin, UK). MagStrep “type3” XT magnetic beads were purchased from IBA Life Sciences (Göttingen, Germany). CA200645 was obtained from HelloBio (Bristol, UK). Adenosine receptor ligands: PSB-603, MRS1220, ZM241385, and DPCPX were sourced from Tocris Bioscience (Bristol, UK). Other reagents were acquired from Sigma-Aldrich (UK).

### cDNA Constructs

We generated NLuc-hA_1_AR as described previously (Stoddart et al., 2015)^3^ by amplifying the full-length sequence of NLuc luciferase (provided by Promega in the pNL1.1. vector) and fusing it in frame with the membrane signal sequence of the 5-HT_3A_ receptor within pcDNA3.1 to yield sig-NLuc. We then fused the full-length human sequence of the A_1_AR (with the methionine start sequence mutated to leucine) to the 3′ end of Sig-NLuc in pcDNA3.1. The NLuc-A_2A_-AR in pcDNA3.1 was generated in a similar way by fusing the full-length human sequence of the A_2A_-AR (with the methionine start sequence mutated to leucine) to the 3′ end of the Sig-NLuc in pcDNA3.1. For the SNAP-A_1_AR cDNA, we fused the full-length human sequence of the A_1_-AR (with the methionine start sequence mutated to leucine) to the 3′end of a Sig-SNAP construct in pcDNA3.1, following removal of the A_3_-AR from a previously described Sig-SNAP-A_3_ AR pcDNA3.1 plasmid (Vernall et al., 2012).^5^ DNA encoding the TwinStrep tag in frame with a SNAP tag, separated by a glycine-serine-serine-glycine linker (with the SNAP start codon mutated to leucine), was purchased from Twist Bioscience (San Francisco, CA, USA). The Twin-Strep-SNAP DNA was ligated in frame into a pcDNA3.1+ Neo A_1_ plasmid, following restriction with enzymes *Kpn*I and *Bam*HI (Promega, Wisconsin, USA) resulting in an expression construct of the TwinStrep-SNAP-A_1_AR (TS-SNAP-A_1_AR) preceded by the signal peptide of the murine 5HT_3A_ receptor.

### Cell Lines

HEK293T cells were purchased from ATCC. HEK293G cells expressing the GloSensor cAMP biosensor (HEKG) were obtained from Promega (Southampton, UK). HEK293 cells stably expressing NLuc-A_1_AR (human), NLuc-A_2B_AR (human), and NLuc-A_3_AR (human) were generated as previously described in Stoddart et al. (2015)^3^ and Comeo et al. (2020).^6^ We generated Twin-Strep-SNAP-A_1_ (human) ARs in HEK239G cells as described in Comeo et al. (2024).^24^ All cells were maintained in DMEM containing 10% FCS and 4 mM l-glutamine at 37 °C in a humidified atmosphere with air/CO_2_ (19:1).

### Transient Transfection

HEK293T cells were transiently transfected with NLuc-A_2A_ (human) AR, NLuc-A_1_ (human) AR, or SNAP-A_1_ (human) AR. HEK293T cells at approximately 80% confluence in T75 flasks were split and seeded into 6-well plates with 40–50 k cells per well in 2 mL. The following day, cDNA (250 ng) was mixed with FuGENE (1:3 ratio) and Optimem to a total volume of 100 μL, incubated for 10 min at room temperature, then added to one well of the 6-well plate. After 24h transfected HEK293T cells were collected and seeded into 96-well plates precoated with Poly-d-lysine at 30–35 k cells per well for NanoBRET experiments. For FLIM and confocal imaging, HEK293T cells at approximately 80% confluence in T75 flasks were split and seeded into 8-well plates (Nunc Lab-Tek, Thermo Fischer Scientific), precoated with Poly-d-lysine, at 7000–12,000 cells per well in 300 μL DMEM/10% FCS. The following day, cDNA (300 ng) was mixed with FuGENE (1:4 ratio) and Optimem to a total volume of 11 μL, incubated for 10 min at room temperature, then added to one well of the 8-well plate. All cells were maintained in DMEM containing 10% FCS and 4 mM l-glutamine at 37 °C in a humidified atmosphere with air/CO_2_ (19:1).

### Nano-BRET Based Ligand-Binding Studies

For saturation binding assays, HEK293 cells stably expressing NLuc-hA_1_ARs were seeded with 30–35k cells per well in 100 μL media into white flat bottomed 96-well Greiner plates (Bio One, UK) precoated with Poly-d-lysine. The next day, the media in the 96-well plates were aspirated and replaced with HEPES buffered saline solution (HBSS: 145 mM NaCl, 5 mM KCl, 1.7 mM CaCl_2_, 1 mM MgSO_4_, 10 mM HEPES, 2 mM sodium pyruvate, 1.5 mM NaHCO_3_, 10 mM d-glucose, pH 7.4). Cells were incubated at 37 °C in humidified air for 30 min in the presence or absence of 1 μM DPCPX before addition of CA200645 or probe **6**. After an hour of incubation, furimazine (Promega) diluted 40 times in HBSS was added to each well (10 μL) and incubated for 5 min at 37 °C. The plate was then read on a PHERAstar FS plate reader (BMG Labtech) at 37 °C. Emissions were read at 450 nm (80 nm bandpass; donor NanoLuc emission) and >610 nm (long pass; fluorescent probe emission) for the BODIPY630/650-labeled probes. A_2A_, A_2B_, and A_3_ AR binding affinity assessments followed similar procedures to that of the A_1_AR. Plates with cells expressing the corresponding NLuc-AR subtypes were prepared, and nonspecific binding was defined by preincubation of cells with 1 μM of subtype-selective antagonists (ZM241385 for A_2A_, PSB603 for A_2B_, and MRS1220 for A_3_). For competition assays, the media in the 96-well plate was aspirated and refilled with 50 μL of 30 nM CA200645 (prepared in HBSS) and increasing concentrations of probe **4** or DPCPX in 50 μL of HBSS. After an hour of incubation at 37 °C in humidified air for equilibrium, furimazine (Promega) diluted 40 times in HBSS was added to each well (10 μL) and incubated for 5 min at 37 °C in humidified air for equilibrium. The plate was then read on a PHERAstar FS plate reader (BMG Labtech) at 37 °C. Emissions were read at 450 nm (80 nm bandpass; donor NanoLuc emission) and >610 nm (long pass; fluorescent probe emission). A_2A_, A_2B_, and A_3_ AR binding affinity assessments followed similar procedures as A_1_AR. Plates with cells expressing corresponding NLuc-AR subtypes were prepared, and nonspecific binding was defined with subtype selective antagonists (ZM241385 for A_2A_, PSB603 for A_2B_, and MRS1220 for A_3_).

### Demonstration of the Availability of the Orthosteric Binding Site Following Ligand-Directed Covalent Labeling Using NanoBRET

To evaluate the availability of the orthosteric binding site following covalent labeling with probe **4**, HEK293T cells transiently expressing NLuc-hA_1_ ARs in 96-well white plates were used. On the day of the experiment, the media was removed, and cells were washed twice with warm HBSS. Cells were then incubated with or without 250 nM of probe **4** in HBSS for 1 h, followed by two washes with HBSS, and incubated with or without 500 nM Tetrazine-AF488 in HBSS for 1 h. Cells were then washed twice with HBSS and incubated with 100 nM of probe **6** in the presence or absence of 1 μM DPCPX for 1 h. To define nonspecific binding cells were preincubated with 1 μM DPCPX for 30 min. Furimazine (Promega), diluted 40 times with HBSS, was added to each well (10 μL per well) followed by a 5 min equilibrium. The plate was read on a PHERAstar FSX plate reader (BMG Labtech) at 37 °C. For red BRET measurement, emissions were read at 450 nm (80 nm bandpass; donor NanoLuc emission) and >610 nm (long pass; fluorescent probe **6** emission). For parallel green BRET measurements, emissions were read at 475 nm (30 nm bandpass; donor NanoLuc emission) and at 535 nm (30 nm bandpass; AF488 tag emission).

### Receptor Purification Following Ligand-Directed Covalent Labeling of the Human A_1_AR with Probe **4**

For receptor purification studies we used HEK293G cells stably expressing the TS-SNAP A_1_AR at 80–90% confluency in T175 flask. For positive controls, the media was aspirated and replaced with 50 nM SNAP-surface AF647 in 10 mL of DMEM. For the experimental sets, the medium was replaced with 10 mL DMEM or DMEM containing 10 μM of DPCPX. The medium was then aspirated and replaced with 200 nM probe **4** in 10 mL of DMEM. After an hour, the media was aspirated, and cells were gently washed with warm PBS twice. 500 nM of Tetrazine-sulfoCy5 in 10 mL of DMEM was added to probe **4** prelabeled cells for an additional 1 h incubation. At the end of incubation, the media was aspirated, and cells were gently washed with 5 mL of PBS twice. Five mL of enzyme-free cell dissociation solution (Sigma-Aldrich) was added to T175 flasks, and cells were detached for 1–2 min. Cells were then collected with 5 mL of PBS and centrifuged at 1,000xRCF for 5 min. Cell pellets were then weighed and resuspended in solubilization buffer (0.5% (w/v) Lauryl Maltose Neopentyl Glycol (LMNG) (Thermo Fisher Scientific, UK), 0.01% (w/v) Cholesteryl Hemisuccinate Tris salt (CHS; Anatrace, OH, USA), 20 mM HEPES, 10% (v/v) glycerol, 150 mM NaCl, complete protease inhibitors (Roche, UK), pH 7.5) at a ratio of 1:10 (w/v) of cell pellet to solubilization buffer. Resuspended cells were solubilized for 2 h on a DigiRoller 6 roller (SLS, UK) at 80 rpm and 4 °C. Samples were then centrifuged at 16,000*g* for 20 min at room temperature and the supernatant collected. Twenty μL of MagStrep “type3” XT magnetic beads (IBA, Göttingen, Germany) were then added to an amber microcentrifuge tube and equilibrated with 200 μL of receptor solubilization buffer twice. 1.2 mL of supernatant from the centrifuged solubilized cells was then added to the magnetic beads and the tube fixed on a head-to-head shaker overnight in a cold room. The next day, the supernatant was removed from the beads using a magnetic separator, and the beads were washed with 200 μL of solubilization buffer twice. The beads were then resuspended with 30 μL of elution buffer (1:9 solution of 10x buffer BXT (IBA Göttingen, Germany) and solubilized buffer) and fixed on a head-to-head shaker for 4 h in a cold room. Samples were then separated from beads using a magnetic separator and then immediately processed for electrophoresis.

### SDS-PAGE Gel Electrophoresis and in Gel Fluorescence

Thirty μL of samples containing purified TS-SNAP-A_1_ were mixed with 10 μL NuPAGE LDS sample buffer and resolved on a NuPage 4–12% Bis-Tris 1 mm x 10 well gel using NuPage MOPS SDS running buffer. Gels were run for 50 min at 200 V. Five μL PageRuler Prestained Protein Ladder was used as the marker. Gels were scanned on an Amersham Typhoon imaging system (GE Healthcare Life Sciences, Pittsburgh, PA) using Fluorstage and Cy5 670BP30 filter sets with PMT set to auto and pixel size set to 200 μm. After acquiring Cy5 scan images, the gel was stained with InstantBlue Coomassie Protein Stain (Abcam) 10 mL overnight. The next day, the gel was washed with Milli-Q water twice to remove excess dye and scanned with the Typhoon imaging system using Fluorstage and IRlong 825BP30 with PMT set to auto and pixel size set to 200 μm.

### Confocal Imaging

HEK293T cells transiently expressing SNAP-hA_1_ARs in an 8-well plate were incubated with 200 μL of 250 nM SNAP-surface AF488 for 30 min. Cells were then washed once with warm DMEM and incubated with DMEM in the presence or absence of 10 μM DPCPX for 30 min. Probe **4** was then added in 20 μL to achieve a final concentration of 100 nM. After 2 h, the media was removed, and the cells were washed twice with warm DMEM, followed by a final incubation with 200 μL of 1 μM Tet-SulfoCy5 for 15 min. At the end of the incubation, the media was removed, the cells were washed twice with warm PBS and then fixed with 4% paraformaldehyde (Sigma-Aldrich) at room temperature for 20 min. In some experiments the cells were washed twice with warm DMEM at the end of the Tet-SulfoCy5 labeling period and incubated with 200 μL of DMEM or 10 μM 2-chloro-*N* ^6^-cyclopentyladenosine (CCPA) for 2 h. At the end of the treatment, the media was removed from the plate and fixed with 4% paraformaldehyde (Sigma-Aldrich) as described above. Fixed cell imaging was conducted using a Zeiss LSM 710 laser scanning confocal microscope fitted with a Zeiss C-Apochromat 40×1.2 NA water immersion objective. A 633 nm HeNe laser was employed for the excitation of the SulfoCy5 fluorophore, and a 488/561/633 dichroic was used for emission detection between 638 and 759 nm. A 488 nm HeNe laser was used to excite AF488, and emission was detected between 492 and 534 nm. The pinhole diameter (1 Airy Unit; 1.1 μm optical slice), laser power, and gain were kept constant in all experiments. Images were acquired at 16 bit depth, 1024 × 1024 pixel resolution with a line averaging of 2 and a pixel dwell time of 3.14 μs. Images were processed in Zeiss ZEN 3.9 (blue edition), and linear adjustments to brightness and contrast were applied equally across all images. To obtain membrane or intracellular intensity values, regions of interest were manually drawn around cell membranes or intracellular regions and measured via FIJI (ImageJ) version 2.16.0 software.

### FLIM-FRET Analysis

Eight-well plates, glass-bottomed ibidi seeded with HEK293T cells transiently expressing NLuc-hA_1_ARs were used for these experiments. On the day of the experiment, the media was removed, and cells were incubated with 250 nM of probe **4** in HBSS for 1 h. Cells were then washed twice with HBSS and labeled with 500 nM of Tetrazine-AF488 in HBSS for 1 h. After two washes with HBSS, cells were further incubated for 1 h in HBSS, 100 nM probe **6** in HBSS, 10 μM DPCPX in HBSS or probe **6** in the presence of 10 μM DPCPX. In the latter case cells were preincubated for 30 min with 10 μM DPCPX in HBSS before addition of probe **6**. Fluorescence lifetime images were captured using a PicoQuant MicroTime200 microscope on an Olympus IX 83 body equipped with a HydraHarp400 TCSPC unit and a 60× water objective, 1.2 NA at 20 °C. Samples were excited with 485 and 638 nm pulsed interleaved lasers (40 MHz) utilizing a 485/640 dichroic and signal collected onto two SPAD detectors with either 535/50 or 670/90 bandpass emission filters. Twenty frames of 256 × 256 pixels were captured at a 10 μs pixel dwell time before being analyzed within Symphotime64 software. The average amplitude-weighted fluorescence lifetime of the donor was calculated from five independent experiments (three of which involved incubation with 10 μM DPCPX), with three replicate fluorescence lifetime images analyzed per experiment.

### FCCS Measurements

Cells were prepared as per the FLIM-FRET experiments with an additional wash procedure, buffer replaced 3 times over a duration of 20 min, prior to data collection. Fluorescence fluctuation trace reads were recorded on a PicoQuant MicroTime200 microscope on an Olympus IX 83 body equipped with a HydraHarp400 TCSPC unit, a 50 μm pinhole and a 60× water objective, 1.2 NA at 20 °C. Samples were excited with 485 and 638 nm pulsed interleaved lasers (40 MHz) utilizing a 485/640 dichroic and signal collected onto two SPAD detectors with either 535/50 or 670/90 bandpass emission filters. Four × 20s trace reads were recorded on the apical membrane at approximately 1.5 kW/cm^2^ laser power, auto- and cross–correlation curves were generated within Symphotime64 software and fit with a single component, 2D diffusion model,^100^ including triplet (limited <20 μs). The cross-correlation particle number (fc) was determined using the following equation^35^

$$fc=\frac{G_{X}\left(0\right)}{\max\left(G_{G}\left(0\right),G_{R}\left(0\right)\right)}$$

where *G*_*x*_(O) is the cross-correlation function at time 0, and *G*_R_(0) and *G*_G_(0) are the autocorrelation functions at time 0 for the red (670/90) and green (535/50) channels, respectively. The precise dimensions of the confocal volume were determined on each experimental date by measuring the diffusion properties of 20 nM ATTO655; taking the diffusion coefficient as *D* = 375 μ ms^–1^ at 20 °C^36^.

### Data Analysis

Data were analyzed using Prism 10.1.1 software (GraphPad, San Diego, USA). Saturation NanoBRET curves were fitted simultaneously for total and nonspecific binding using the following equation

$$total\;binding=\frac{B_{\max}\times \left[B\right]}{\left[B\right]+K_{D}}+m\times B+c$$

where *B*_max_ is the maximal specific binding, [*B*] is the concentration of the fluorescent ligand (nM), *K*_*D*_ is the equilibrium dissociation constant (nM), *m* is the slope of the nonspecific binding component, and *C* is the *y*-axis intercept.

The affinities of ligands at the various adenosine receptors were calculated from competition binding data with a one-site sigmoidal response curve given by the following equation

$$\%\;Inhibition\;of\;specific\;binding=\frac{\left(100\times \left[A^{n}\right]\right)}{\left(\left[A^{n}\right]+{IC}_{50}^{n}\right)}$$

where [*A*] is the concentration of unlabeled ligand, *n* is the Hill coefficient, and IC_50_ is the concentration of ligand required to inhibit 50% of the specific binding of the CA200645. The IC_50_ values were then used to calculate the *K*_*i*_ values using the Cheng-Prusoff equation

$$K_{i}=\frac{{IC}_{50}}{1+\frac{\left[L\right]}{K_{D}}}$$

where [*L*] is the concentration of CA200645 in nM, and *K*_D_ is the dissociation constant of that fluorescent ligand in nM. The *K*_D_ values for CA200645 determined for the four adenosine receptor subtypes were 15 nM for A_1_, 30 nM for A_2A_, 10 nM for A_2B_, and 30 nM for A_3_ AR.

## Supplementary Material





## Notes

Supporting Figures, Schemes and characterization data of the compounds described (PDF)
Molecular formula strings of tested compounds (CSV)

## Abbreviations

A_1_AR: A_1_ adenosine receptor
BEP: 2-bromo-1-ethyl-pyridinium tetrafluoroborate
COMU: (1-cyano-2-ethoxy-2-oxoethylidenaminooxy)­dimethylamino-morpholino-carbenium hexafluorophosphate
DMF: dimethylformamide
LDLC: ligand-directedly covalent labeling.
