# Supplementary material for: Two-Step Ligand-Directed Covalent Fluorescent Labeling of the Adenosine A1‑Receptor That Maintains Its Orthosteric Binding Site’s Availability to Bind Ligands
Source: J Med Chem. 2026 Jan 8;69(3):2481–95. doi: 10.1021/acs.jmedchem.5c02389 (PMC12910660; doi:10.1021/acs.jmedchem.5c02389)

Supporting Information

Two-step ligand-directed covalent fluorescent labeling of the adenosine A_1-receptor that maintains its orthosteric binding site's availability to bind ligands.

Chia-Yang Lin[1,2], *Simon Platt*[2,3], *Joelle Goulding*[2,3], *Stephen J Briddon*[2,3], *Nicholas D Kindon*[1,2], *Clare R. Harwood*[2,3,] *Chih-Wei Lai*[4], *Barrie Kellam*[1,2, *] & *Stephen J Hill*[2,3,*]

[1] School of Pharmacy, Division of Bimolecular Science and Medicinal Chemistry, Biodiscovery Institute, University of Nottingham, Nottingham, NG7 2RD, United Kingdom.

[2] Centre of Membrane Proteins and Receptors (COMPARE), University of Birmingham and University of Nottingham, The Midlands, NG7 2UH, United Kingdom.

[3] School of Life Sciences, Division of Physiology, Pharmacology and Neuroscience University of Nottingham, Nottingham, NG7 2UH, United Kingdom.

[4] School of Pharmacy, National Defense Medical University, Taipei, 114201, Taiwan

Corresponding Authors

Barrie Kellam: barrie.kellam@nottingham.ac.uk

Stephen Hill: stephen.hill@nottingham.ac.uk

Contents

Supplementary Table 1. LDCL probes (transfer sulfoCy5) binding affinity across four adenosine subtype receptors ....S**Error! Bookmark not defined.**

Supplementary Figure 1. Specific binding curves of NL-A_1AR.. ....S4

Supplementary Figure 2. Pharmacological properties of probe **6**...S5

Supplementary Figure 3. Availability of the orthosteric binding pocket to bind probe **6** after prior LDCL labeling with probe **4** and tetrazine-AF488.. ....S6

Supplementary Scheme 1. Probe **6** and TCO cargo synthesis. S7

Compound synthesis and characterization. S8

Supplementary Figure 4. HRMS (top) and analytical RP-HPL Chromatogram (bottom) of probe **4**.. ....S13

Supplementary Figure 5. HRMS (top) and analytical RP-HPL Chromatogram (bottom) of probe **6**.. ....S14

NMR SPECTRA. S15

Supplementary Table 1. LDCL probes (transfer sulfoCy5) binding affinity across four adenosine subtype receptors.

Ligand	Structure information			$K_D \pm$ SEM (nM) [a]				A_1/A_{2A} ratio
	Linker	X	Y	NL-hA_1	NL-hA_{2A}	NL-hA_{2B}	NL-hA_3	
S1	β-Alanine	F	R1	45.51 ± 9.95	267.90 ± 113.14	>500 [b]	>500 [b]	5.9 [c]
S2	Glycine	F	R1	8.40 ± 3.75	158.64 ± 14.50	>500 [b]	>500 [b]	18.9 [c]
S3	Glycine	R1	F	28.26 ± 5.92	261.46 ± 57.14	>500 [b]	>500 [b]	9.3 [c]
S4	GABA	F	R1	33.64 ± 13.05	276.96 ± 34.73	>500 [b]	>500 [b]	8.2 [c]
S5	GABA	R1	F	28.50 ± 8.96	387.74 ± 78.56	>500 [b]	>500 [b]	13.6 [c]

a. Dissociation constants (K_D) were determined using NanoBRET-based saturation binding assays. Each adenosine receptor (AR) subtype was assessed in five independent experiments, each performed in triplicate. A_1, A_{2B}, and A_3 ARs with N-terminal NanoLuc (NL) tags were stably expressed in HEK293 cells, while A_{2A} AR with an N-terminal NL tag was transiently expressed in HEK293T cells. Ligand concentrations ranged from 0 to 500 nM and were incubated with cells for 1 hour at 37 °C. Non-specific binding was defined by preincubation with 1 µM of a subtype-selective antagonist for 30 min at 37 °C: DPCPX (A_1), ZM241385 (A_{2A}), PSB603 (A_{2B}), and MRS1220 (A_3).

b. For cases where total binding nearly overlapped with non-specific binding at concentrations up to 500 nM, K_D values are reported as >500 nM, as higher concentrations were not tested.

c. Selectivity ratios were calculated as $K_D(A_{2A})/K_D(A_1)$.

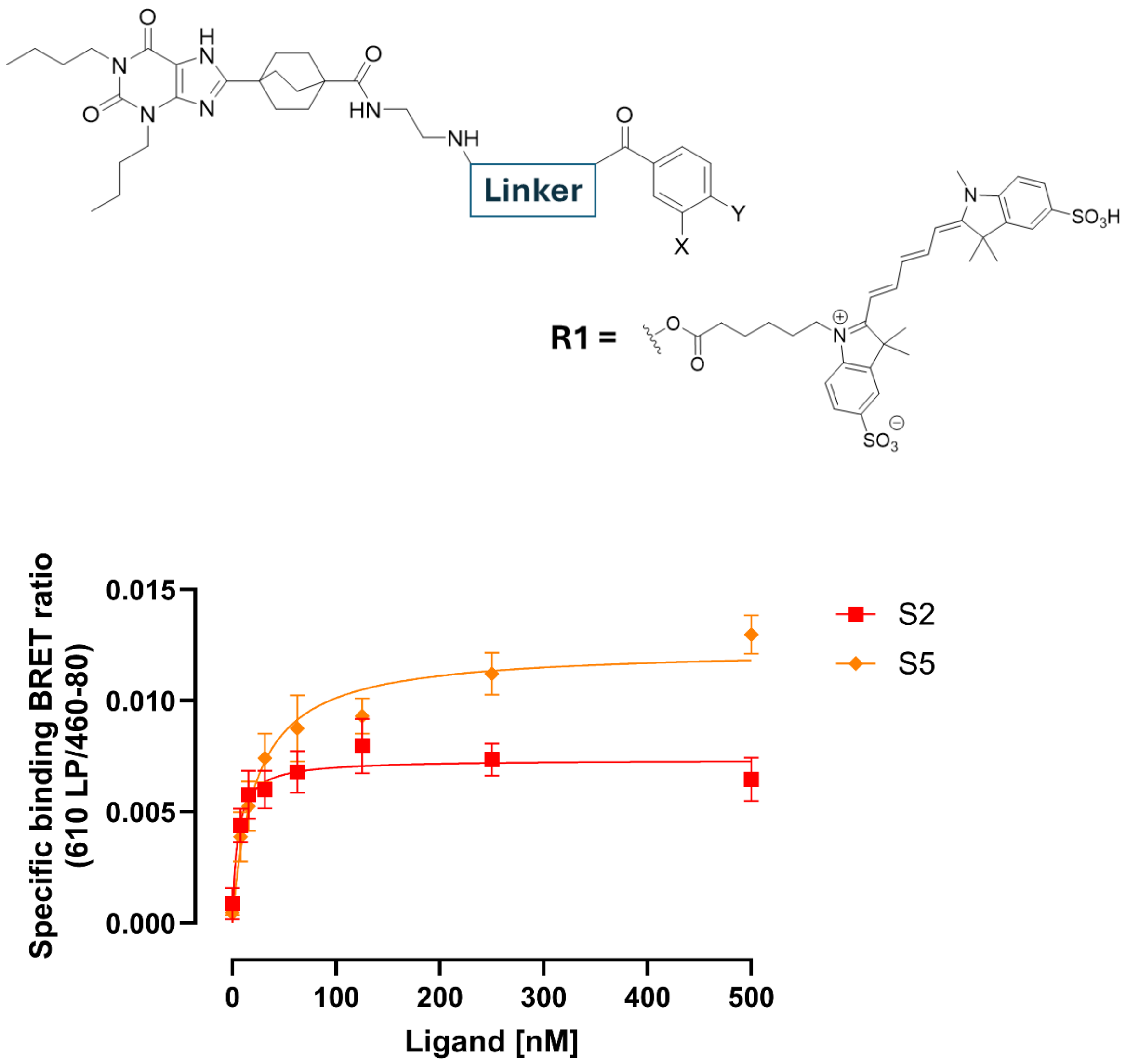

Supplementary Figure 1. Specific binding curves of NL-A_1AR. Compounds S2 and S5 exhibit higher A_1/A_{2A} selectivity among the ligands listed in Supplementary Table 1. Analysis of the A_1AR-specific binding curves shows that S5 produces a higher signal intensity compared to S2, suggesting superior cargo transfer efficiency. Specific binding was calculated by subtracting non-specific binding from total binding, based on five independent saturation binding assays performed in triplicate. Non-specific binding was defined as the signal from cells preincubated with 1 µM DPCPX for 30 minutes. The incubations in saturation assays and subsequent plate readings were conducted at 37 °C.

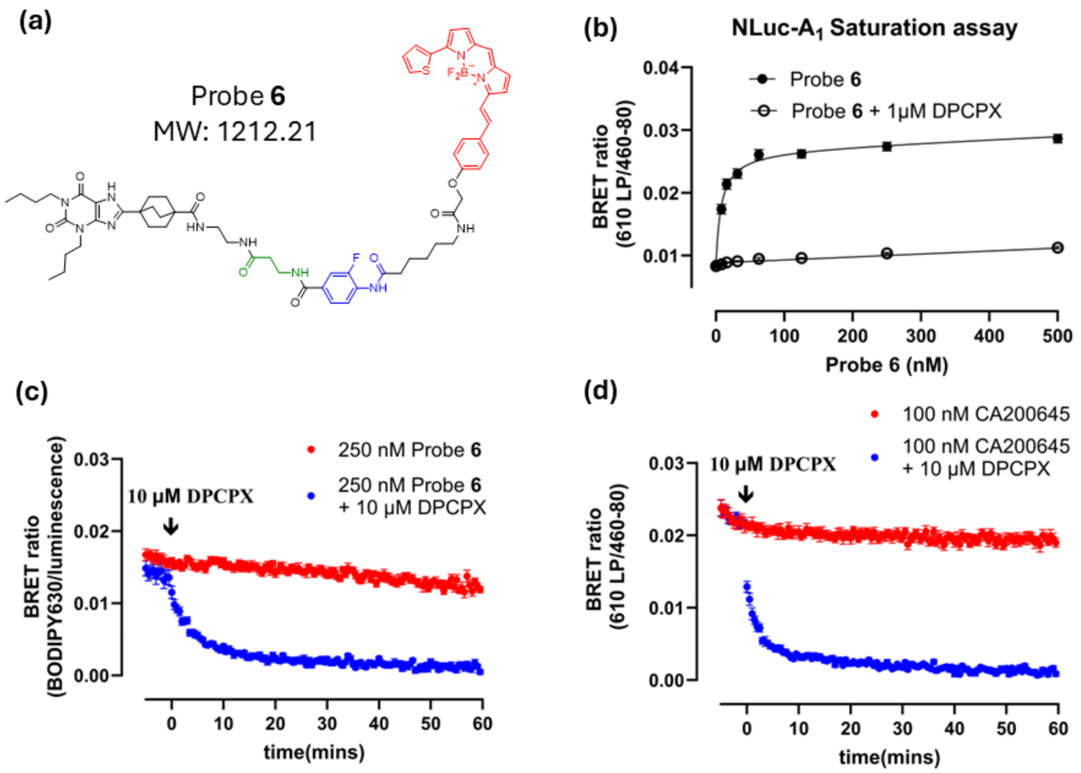

Supplementary Figure 2. Pharmacological properties of probe **6**. (a) Structure of probe **6**. Note that probe **6** contains the BODIPY 630/650 fluorophore (highlighted in red) and contains a 2-fluorophenyl amide group (highlighted in blue) instead of a 2-fluorophenyl ester, and therefore does not covalently transfer cargo to the A_1AR receptor. (b) Total and non-specific binding of probe **6** to HEK293 cells stably expressing NL-A_1AR. The x-axis represents probe **6** concentration (nM), while the y-axis shows the BRET ratio, calculated as the signal above 610 nm divided by the signal from 420-500 nm. Non-specific binding was assessed by preincubating cells with 1 µM DPCPX for 30 minutes before addition of probe **6**. (c), (d) NLuc-A_1AR dissociation assays. HEK293 cells stably expressing NL-A_1AR were incubated with (c) 250 nM probe **6** or (d) 100 nM CA200645 for 2h at 37ºC, followed by furimazine addition. After a five-minute baseline measurement, 10 µM DPCPX was added and BRET measurements continued for a further 60 min (one measurement every 30 seconds) at 37ºC.

Data represent specific binding, calculated by subtracting non-specific binding (from wells pretreated with 10 µM DPCPX) from total binding. Values are expressed as mean ± SEM from five independent dissociation assays.

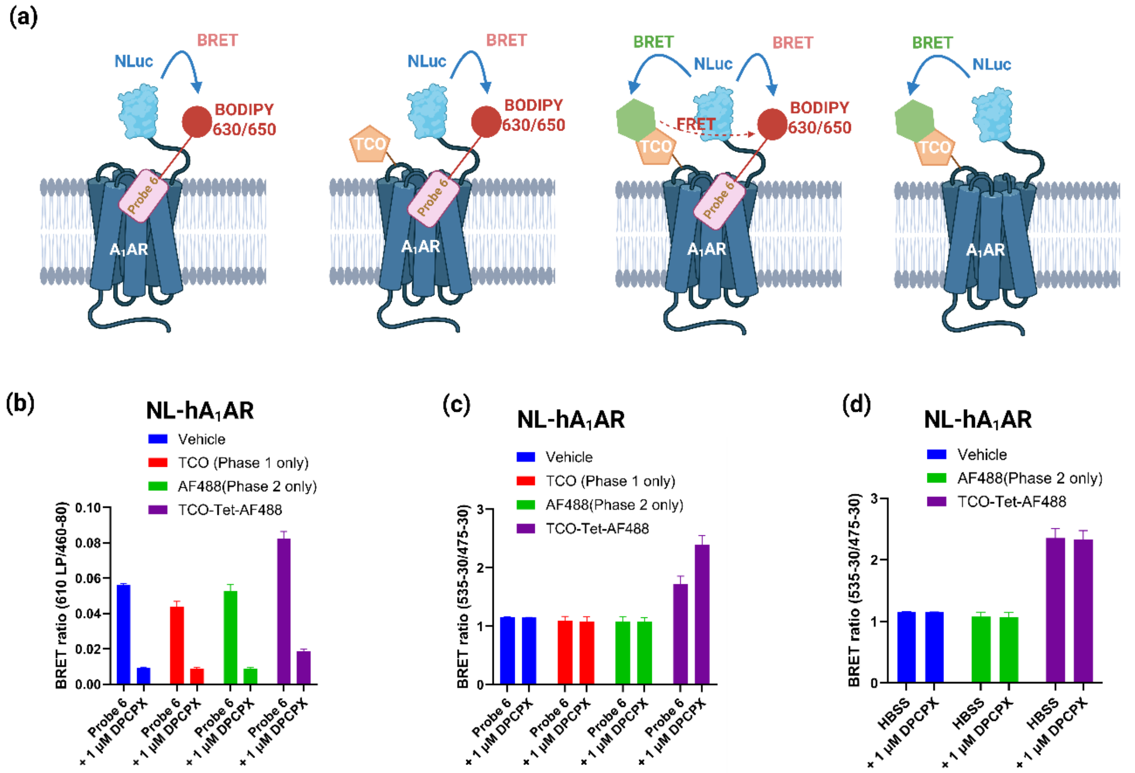

Supplementary Figure 3. Availability of the orthosteric binding pocket to bind probe **6** after prior LDCL labeling with probe **4** and tetrazine-AF488. (a) Schematics showing the different configurations for NanoBRET and FRET on binding of probe **4** under control conditions and following pre-labeling of HEK293T cells transiently expressing the human NL-A_1AR with (250 nM) probe **4** to attach a TCO group and interaction with 500 nM tetrazine-AF488. (b) NanoBRET signals generated in the red channel following addition of 100 nM probe **6**. The vehicle represents experiments conducted on HEK293T cells expressing the NL-A_1AR without covalent labeling with probe **4** and tetrazine-AF488 in the presence and absence of 1 µM DPCPX. AF488 tagging requires two steps: (1) covalent transfer of the TCO group to the NL-

A$_1$AR and (2) a click reaction with Tet-AF488. Conditions containing either probe **4** alone (TCO phase 1), Tet-AF488 alone (AF488 phase 2) or the full two-step LDCL labeling process (TCO-tet-AF488) are also included. (c) Measurements made in the green channel for the same conditions as in (b). (d) A control set for green BRET measurements made in the presence of HBSS without probe **6**. All data represent mean ± SEM from five independent experiments. Created in BioRender. Lai, C. (2026) https://BioRender.com/rhksiks

Supplementary Scheme 1. Probe **6** and TCO cargo synthesis [a]

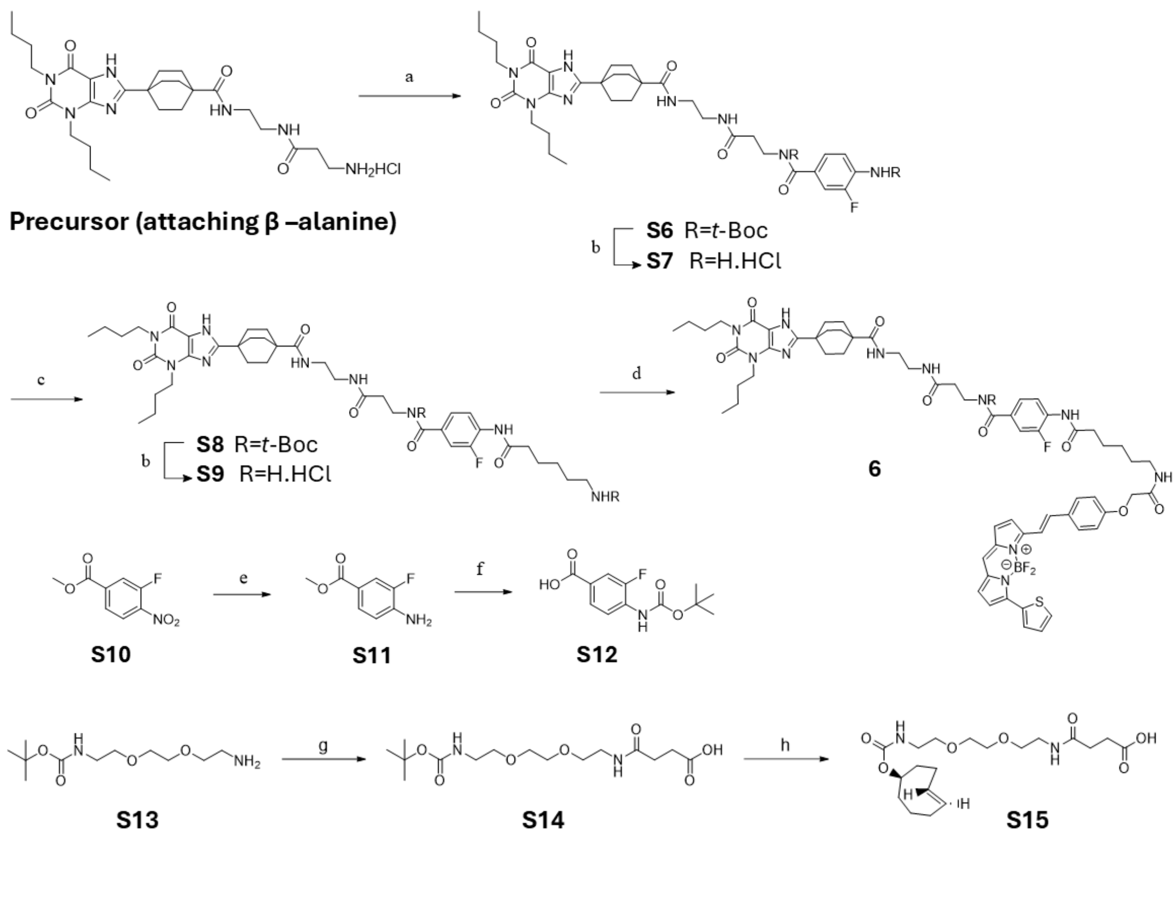

[a] Reagents and conditions: (a) 4-((*tert*-butoxycarbonyl)amino)-3-fluorobenzoic acid (**S12**), COMU, DIPEA, DMF, 80ºC, overnight, 71%; (b) 4 N HCl in dioxane, rt, 1 hr; (c) T3P, DIPEA, Boc-E-ACP-OH, DCM, reflux, 19 hr, 5%;(d) BODIPY630/650 carboxylic acid, BEP, DIPEA, rt, overnight, 35%; (e) Pd/C, H_2, MeOH, rt, 1.5 hr, 78%; (f) 1. DMAP, Boc_2O, THF, reflux, overnight; 2. K_2CO_3, MeOH, reflux, 3 hr; 3. 2M NaOH, MeOH/THF, rt, 5.5 hr, 64%; (g) succinic anhydride, DIPEA, $CHCl_3$, rt, overnight, 36%; (h) 1. 4N HCl in dioxane, rt, 1 hr; 2. TCO-NHS ester, DIPEA, rt, overnight, 63%.

Compound synthesis and characterization

***tert*-butyl (4-((3-((2-(4-(1,3-dibutyl-2,6-dioxo-2,3,6,7-tetrahydro-1*H*-purin-8-yl)bicyclo[2.2.2]octane-1-carboxamido)ethyl)amino)-3-oxopropyl)carbamoyl)-2-fluorophenyl)carbamate (S6)**

Crude hydrochloride salt of **Precursor attaching β-alanine** (Comeo *et al.* 2024)[1] (178 mg, 0.32 mmol, 1 eq) was reacted with **S12** (97 mg, 0.38 mmol, 1.2 eq) following General Procedure A. The reaction was heated to 80°C and stirred overnight. The reaction was monitored by LC-MS. The work-up method was used as no precipitate formed when water was added to the mixture. Purification was conducted *via* automated flash column chromatography (gradient MeOH: DCM from 2:98 to 10:90, 17 CV) and afforded compound **S6** (175 mg, 0.228 mmol, yield = 71.3%). LC-MS m/z calcd. for $C_{39}H_{55}FN_8O_7$ [M-H$^+$] 767.425; found 767.2, t_R = 2.87 min, Method A. ^{1}H NMR (CD_3OD) δ 8.06 (t, J = 8.8 Hz, 1H), 7.63 (m, 2H), 4.12 (t, J = 7.3 Hz, 2H), 3.99 (t, J = 7.3 Hz, 2H), 3.65 (t, J = 6.7 Hz, 2H), 3.31 (s, 4H), 2.52 (t, J = 6.7 Hz, 2H), 2-1.96 (m, 6H), 1.88-1.83 (m, 6H), 1.74 (p, J = 7.4 Hz, 2H), 1.62 (p, J = 7.7 Hz, 2H), 1.53 (s, 9H), 1.39 (m, 4H), 0.97 (t, J = 7.32 Hz, 3H), 0.96 (t, J = 7.44 Hz, 3H). ^{13}C NMR (CD_3OD) δ 178.54, 172.96, 166.15, 160.54, 156.14, 155.33, 152.01, 151.37 (d, J = 243.4 Hz), 148.66, 130.18 (d, J = 10.3 Hz), 128.54 (d, J = 6.1 Hz), 123.26 (d, J = 2.5 Hz), 119.15, 114.09 (d, J = 20.7 Hz), 106.88, 81.68, 43.42, 41.43, 40.23, 40, 38.94, 36.40, 35.86, 33.63, 30.24, 30.12, 29.94, 28.36, 28.22, 20.29, 19.91, 13.88, 13.77.

***N*-(2-(3-(4-amino-3-fluorobenzamido)propanamido)ethyl)-4-(1,3-dibutyl-2,6-dioxo-2,3,6,7-tetrahydro-1*H*-purin-8-yl)bicyclo[2.2.2]octane-1-carboxamide hydrochloride salt (S7)**

Compound **S6** (60 mg, 78 μmol, 1 eq) was synthesized using General Procedure C. LC-MS was used to monitor the reaction. Once the *t*-Boc group was removed, the mixture was evaporated to afford crude compound **S7** for the next step without further purification.

***tert*-butyl (6-((4-((3-((2-(4-(1,3-dibutyl-2,6-dioxo-2,3,6,7-tetrahydro-1*H*-purin-8-yl)bicyclo[2.2.2]octane-1-carboxamido)ethyl)amino)-3-oxopropyl)carbamoyl)-2-fluorophenyl)amino)-6-oxohexyl)carbamate (S8)**

Crude compound **S7** obtained from the *t*-Boc deprotection of compound **S6** (60 mg, 78 µmol, 1 eq), BoC-E-Acp-OH (20.7 mg, 89.7 µmol, 1.15 eq), propylphosphonic anhydride (T3P) (0.16 mL, 269.1 µmol, 3.45 eq), and DIPEA (78 µL, 448.5 µmol, 5.75 eq) were dissolved in DCM. The reaction was refluxed and stirred for 19 hr. The reaction was monitored by LC-MS. The work-up method was used as no precipitate formed when water was added to the mixture. Purification was conducted *via* automated flash column chromatography (gradient EtOAc: Cyclohexane from 25:75 to 100:0, 20 CV; followed by gradient MeOH: DCM from 10:90 to 20:80, 10 CV) and afforded compound **S8** (3.7 mg, 4.2 µmol, yield = 5.4%). LC-MS m/z calcd. for $C_{45}H_{66}FN_9O_8$ [M-H$^+$] 880.51; found 879.9, t_R = 2.85 min, Method A. ^{1}H NMR (CD_3OD) δ 8.14 (t, J = 7.8 Hz, 1H), 7.68-7.60 (m, 2H), 4.09 (t, J = 7 Hz, 2H), 3.97 (t, J = 7.5 Hz), 3.64 (t, J = 7.1 Hz, 2H), 3.29 (s, 4H), 3.04 (t, J = 7.3 Hz, 2H), 2.50 (t, J = 7.3 Hz), 2.45 (t, J = 7.9 Hz, 2H), 2-1.91 (m, 6H), 1.88-1.81 (m, 6H), 1.71 (m, 4H), 1.60 (p, J = 7.8 Hz), 1.50 (p, J = 7.3 Hz), 1.42 (s, 9H), 1.40-1.28 (m, 6H), 0.97 (t, J = 7.36 Hz, 3H), 0,96 (t, J = 7.36 Hz, 3H). ^{13}C NMR (CD_3OD): δ 180.49, 174.91, 174.41, 168.2, 162.18, 158.56, 155.98, 154.50 (d, J = 243Hz), 152.81, 149.46, 132.09 (d, J = 6.8Hz), 130.72 (d, J = 11.4Hz), 124.34 (d, J = 3.3Hz), 124.26, 115.5 (d, J = 21.7Hz), 108.21, 79.81, 44.23, 42.15, 41.17, 40.58, 40.18, 40.10, 37.78, 37.43, 36.86, 34.91, 31.25, 31.22, 30.99, 30.71, 29.21, 28.78, 27.41, 26.38, 21.16, 20.83, 14.19, 14.12.

***N*-(2-(3-(4-(6-aminohexanamido)-3-fluorobenzamido)propanamido)ethyl)-4-(1,3-dibutyl-2,6-dioxo-2,3,6,7-tetrahydro-1*H*-purin-8-yl)bicyclo[2.2.2]octane-1-carboxamide hydrochloride salt (S9)**

Compound **S8** (3.5 mg, 4 μmol, 1 eq) was synthesized using General Procedure C. LC-MS was used to monitor the reaction. Once the *t*-Boc group was removed, the mixture was evaporated to afford crude compound **S9** for the next step without further purification.

(*E*)-4-(1,3-dibutyl-2,6-dioxo-2,3,6,7-tetrahydro-1*H*-purin-8-yl)-*N*-(2-(3-(4-(6-(2-(4-(2-(5,5-difluoro-7-(thiophen-2-yl)-5*H*-4l4,5l4-dipyrrolo[1,2-c:2',1'-f][1,3,2]diazaborinin-3-yl)vinyl)phenoxy)acetamido)hexanamido)-3-fluorobenzamido)propanamido)ethyl)bicyclo[2.2.2]octane-1-carboxamide (6)

Crude compound **S9** obtained from the *t*-Boc deprotection of compound **S8** (3.5 mg, 4 μmol, 1 eq) was coupled with BODIPY630/650 carboxylic acid (1.8 mg, 4 μmol, 1 eq) *via* BEP (1.1 mg, 4 μmol, 1 eq) and DIPEA (6 drops) in DMF (0.7 mL). The reaction was carried out at rt and protected from light exposure. After overnight reaction, DMF was removed by rotavapor. The residue was reconstituted with MeCN and MeOH. Further purification was done with RP-HPLC using a YMC C8 semi-preparative column to afford compound **6** (1.7 mg, 1.4 μmol, yield = 35%). HR-MS (TOF-ES$^+$) calcd. m/z for $C_{6\ 3}H_{7\ 3}BF_3N_{1\ 1}O_8S$ [M+Na$^+$] 1234.530165; found 1234.5284, error within 2.4 ppm; Method B. RP-HPLC with a YMC C8 analytic column showed retention time as 27.9 min over 53 min analysis, purity: 97%.

Methyl 4-amino-3-fluorobenzoate (S11)

Compound **S10** (0.8 g, 4 mmol, 1 eq) and Pd/C (0.08 g, 0.1 eq) were dissolved in MeOH (10 mL). Two balloons of H_2 were purged into the flask, and the mixture was stirred at rt for 1.5 hr. The reaction was monitored by TLC. The mixture was filtered through a celite cake, and MeOH was used to wash the cake several times. Collected MeOH was evaporated to afford compound **S11** (0.54 g, 3.19 mmol, yield = 78.4%). LC-MS m/z calcd. for $C_8H_9FNO_2$ [M-H$^+$] 170.05; found 170.1, t_R = 2.55 min, Method A. **^{1}H NMR (DMSO-*d*$_6$)** δ 7.53 (dd, *J* = 8.44, 2.02 Hz, 1H), 7.48 (dd, *J* = 12.24, 2.02 Hz, 1H), 6.78 (t, *J* = 8.64 Hz, 1H), 6.07 (s, 2H), 3.76 (s, 3H).

^{13}C NMR (DMSO-d_6) δ 166.09, 149.61 (d, J = 236.14 Hz), 142.23 (d, J = 12.89 Hz), 127.29 (d, J = 2.6 Hz), 116.43 (d, J = 6.06 Hz), 116.10 (d, J = 19.33 Hz), 115.23 (d, J = 4.93 Hz), 52.0.

4-((*tert*-Butoxycarbonyl)amino)-3-fluorobenzoic acid (S12)

Compound **S11** (845.8 mg, 5 mmol, 1 eq), DMAP (61 mg, 0.5 mmol, 0.1 eq), and Boc_2O (6.55 g, 30 mmol, 6 eq) were dissolved in THF (50 mL). The mixture was refluxed overnight. THF solvent was removed *via* rotavapor. The residue and K_2CO_3 (2 g, 15 mmol, 3 eq) were dissolved in MeOH (50 mL). The mixture was refluxed again for 3 hr. After the mixture cooled to rt, MeOH was removed *via* rotavapor. The residue was reconstituted with EtOAc and extracted with water. The collected EtOAc solution was washed with 2 N HCl twice, saturated $NaHCO_3$, and brine, sequentially. The organic solution was evaporated, and the crude product was used for the next reaction. The crude product and 2 M NaOH (25 mL) were dissolved in 50 mL of organic solvent (MeOH/THF 1:1). The mixture was stirred at rt for 5.5 hr. The organic solvent was removed *via* rotavapor. The residue in water was extracted with EtOAc. The collected aqueous portion was acidified with 2 N HCl to afford a white precipitate. The precipitate was collected *via* filtration and dried in the oven to afford compound **S12** (0.98 g, 3.85 mmol, yield = 64.2%). LC-MS m/z calcd. for $C_{12}H_{14}FNO_4$ [M-H$^+$] 255.09; found 256.1, t_R = 2.73 min, Method A. **^{1}H NMR (DMSO-d_6)** δ 13.03 (s, 1H), 9.36 (s, 1H), 7.90 (t, J = 8.2 Hz, 1H), 7.72 (dd, J = 8.57, 1.88 Hz, 1H), 7.65 (dd, J = 11.45, 1.88 Hz, 1H), 1.48 (s, 9H). **^{13}C NMR (DMSO-d_6)** δ 166.55 (d, J = 2.53 Hz), 153, 152.8 (d, J = 246.08 Hz), 131.66 (d, J = 11.39 Hz), 126.61 (d, J = 6.09 Hz), 126.19 (d, J = 3.28 Hz), 122.48, 116.49 (d, J = 20.8 Hz), 80.52, 28.43.

2,2-Dimethyl-4,15-dioxo-3,8,11-trioxa-5,14-diazaoctadecan-18-oic acid (S14)

Commercially available *tert*-butyl (2-(2-(2-aminoethoxy)ethoxy)ethyl)carbamate (**S13**) (218 mg, 0.87 mmol, 1 eq) was dissolved in 7 mL of $CHCl_3$ and cooled to 0°C in an ice bath. Succinic anhydride (87.6 mg, 0.87 mmol, 1 eq) was added to the cooled **S13** solution, allowing

10 minutes of mixing. The ice bath was removed, and the reaction continued for 1 hr. The $CHCl_3$ was evaporated under reduced pressure, and the crude product was purified through automated flash column chromatography (gradient MeOH: DCM from 1:99 to 10:90, 23 CV) to afford compound **S14** (111 mg, 0.32 mmol, yield = 36%). **^{1}H NMR (400 MHz, $CDCl_3$-*d*)** δ 7.44 (s, 1H, NH), 6.94 (s, 1H, NH), 3.62 (s, 4H), 3.54 (p, *J* = 4.9 Hz, 4H), 3.45 (q, *J* = 5.1 Hz, 2H), 3.32 (q, *J* = 5.2 Hz, 2H), 2.70-2.64 (t, *J* = 5.8 Hz, 2H), 2.5 (t, *J* = 5.6 Hz, 2H), 1.45 (s, 9H). **^{13}C NMR (101 MHz, $CDCl_3$-*d*)** δ 177.64, 173.13, 158.15, 81.26, 77.36, 70.44, 70.35, 69.73, 41.73, 39.45, 31.64, 30.44, 28.50.

(*R*,*E*)-1-(cyclooct-4-en-1-yloxy)-1,12-dioxo-5,8-dioxa-2,11-diazapentadecan-15-oic acid (S15)

Compound **S14** (38.28 mg, 110 μmol, 1.1 eq) underwent *t*-Boc deprotection following General Procedure C. The resulting **S14** hydrochloride salt was coupled with TCO-NHS ester (26.73 mg, 100 μmol, 1 eq) in the presence of 0.2 mL of DIPEA and 1 mL of DMF. The reaction proceeded at rt overnight. 15 mL of Milli-Q water was added to the mixture. The solution was basified with TEA and extracted with EtOAc twice. The aqueous fraction was then acidified with 6% acetic acid (to pH 3-4) and extracted with DCM three times. The collected DCM fraction was dried with anhydrous Na_2SO_4, filtered, and evaporated to dryness. The crude product was further purified through automated flash column chromatography (gradient MeOH: DCM from 2:98 to 10:90, 25 CV) to afford compound **S15** (25.2 mg, 63 μmol, yield = 63%). **^{1}H NMR (400 MHz, CD_3OD)** δ 5.60 (ddd, *J* = 15.2, 9.1, 5.2 Hz, 1H), 5.48 (ddd, *J* = 15.7, 10.7, 3.5 Hz, 1H), 4.37-4.25 (m, 1H), 3.61 (s, 4H), 3.57-3.48 (m, 4H), 3.36 (t, *J* = 5.5 Hz, 2H), 3.26 (t, *J* = 5.7 Hz, 2H), 2.59 (t, *J* = 7 Hz, 2H), 2.48 (t, *J* = 6.9 Hz, 2H), 2.39-2.25 (m, 3H), 2.02-1.86 (m, 4H), 1.79-1.65 (m, 2H), 1.63-1.55 (m, 1 H). **^{13}C NMR (101 MHz, CD_3OD)** δ 176.17, 174.74, 158.63, 135.98, 133.80, 81.79, 71.30, 71.28, 71.04, 70.59, 42.19, 41.55, 40.40, 39.64, 35.19, 33.48, 32.12, 31.53, 30.29.

+MS, 0.7-0.9min #41-52

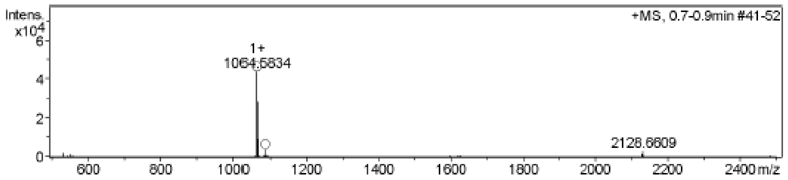

#	m/z	I %
1	532.7953	4.3
2	533.2962	3.0
3	551.7684	2.9
4	552.2695	2.0
5	559.7555	2.1
6	1064.5834	100.0
7	1065.5843	64.5
8	1066.5888	21.4
9	1067.5829	6.0
10	1083.5524	2.0
11	1084.0547	2.5
12	1086.5583	9.3
13	1087.5588	6.0
14	1088.5694	2.2
15	1091.5481	2.0
16	1092.0437	2.4
17	2127.6705	3.7
18	2128.6609	6.8
19	2129.6364	4.8
20	2130.6322	2.0

Generate Molecular Formula Parameters

Charge	Tolerance	sigma limit	H/C Ratio	Electron Conf.	Nitrogen Rule	Chrom.BackGround	Calibration
+1	6 ppm	0.08	3 - 0	both	false	false	TRUE

Expected Formula C54 H78 F1 N9 O12 **Adduct(s):** H, Na, NH4, C3H5N2, radical

#	meas. m/z	theo. m/z	|Err|[ppm]	Sigma	Formula	Adduct	Adduct Mass
1	1 064.5834	1 064.5827	0.70	0.0089	C54H79FN9O12	M+H	1.0078
1	1 086.5583	1 086.5646	5.90	0.0127	C54H78FN9NaO12	M+Na	22.9898

Note: Sigma fits < 0.05 indicates high probability of correct MF

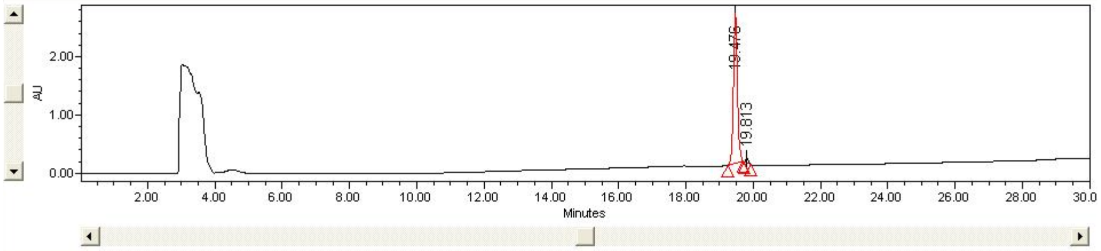

	Name	Retention Time (min)	Purity1 Angle	Purity1 Threshold	PDA Match1 Spect. Name	PDA Match1 Angle	PDA Match1 Threshold	PDA Match1 Lib. Name	Area (µV*sec)	% Area	Height (µV)	Int Type	Amount	Units	Peak Type	Peak
1		19.476							20498735	97.67	2557791	bb			Unknown	
2		19.813							488027	2.33	84356	bb			Unknown	

Supplementary Figure 4. HRMS (top) and analytical RP-HPL Chromatogram (bottom) of probe **4**.

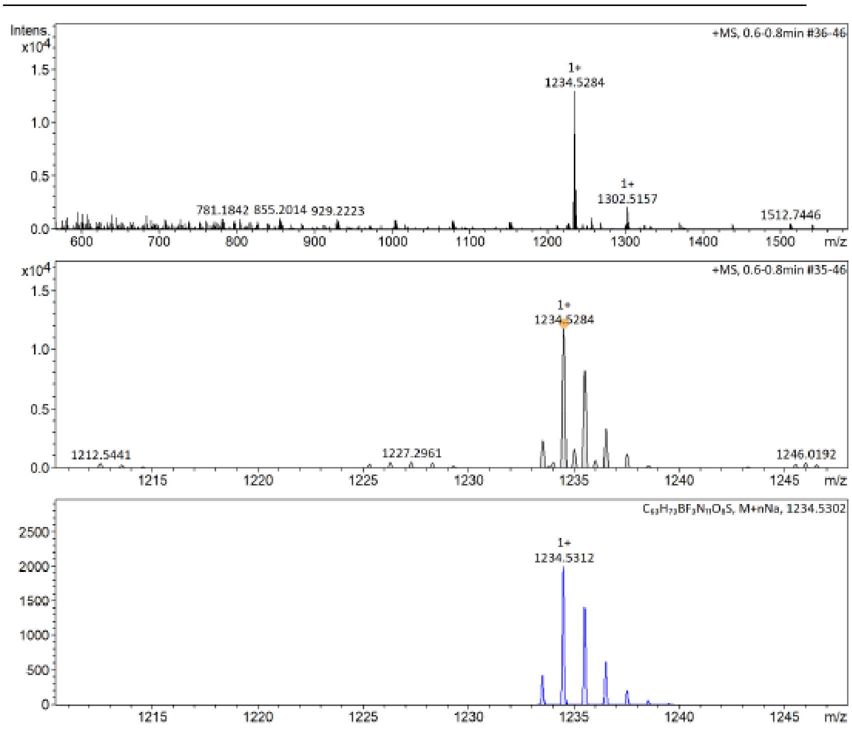

Meas. m/z	#	Ion Formula	Score	m/z	err [mDa]	err [ppm]	mSigma
1234.528419	1	C63H73BF3N11NaO8S	100.00	1234.530165	1.7	1.4	9.4

Note: Sigma fits < 0.05 indicates high probability of correct MF

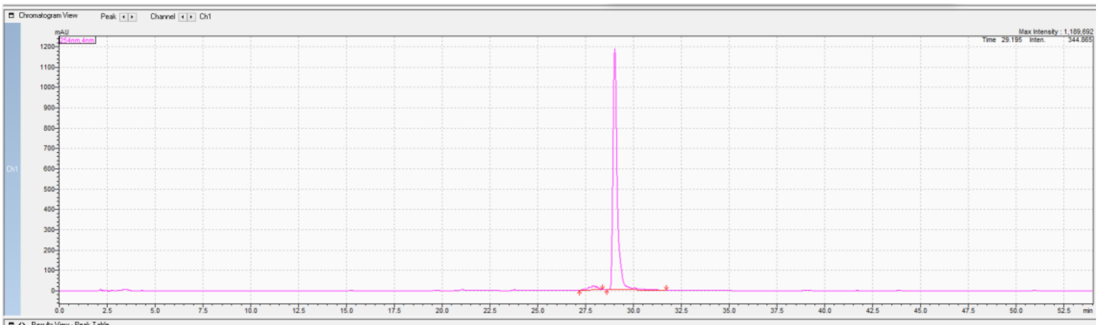

Peak#	Ret. Time	Area	Height	Mark	Conc.	Unit	ID#	Name	Area%
1	27.913	629913	20870	M	0.000				3.309
2	29.035	18405452	1185066	M	0.000				96.691
Total		19035365	1205937		0.000				100.000

Supplementary Figure 5. HRMS (top) and analytical RP-HPL Chromatogram (bottom) of probe **6**.

NMR SPECTRA

^{1}H NMR (400 MHz, DMSO-*d*$_6$) of **1**

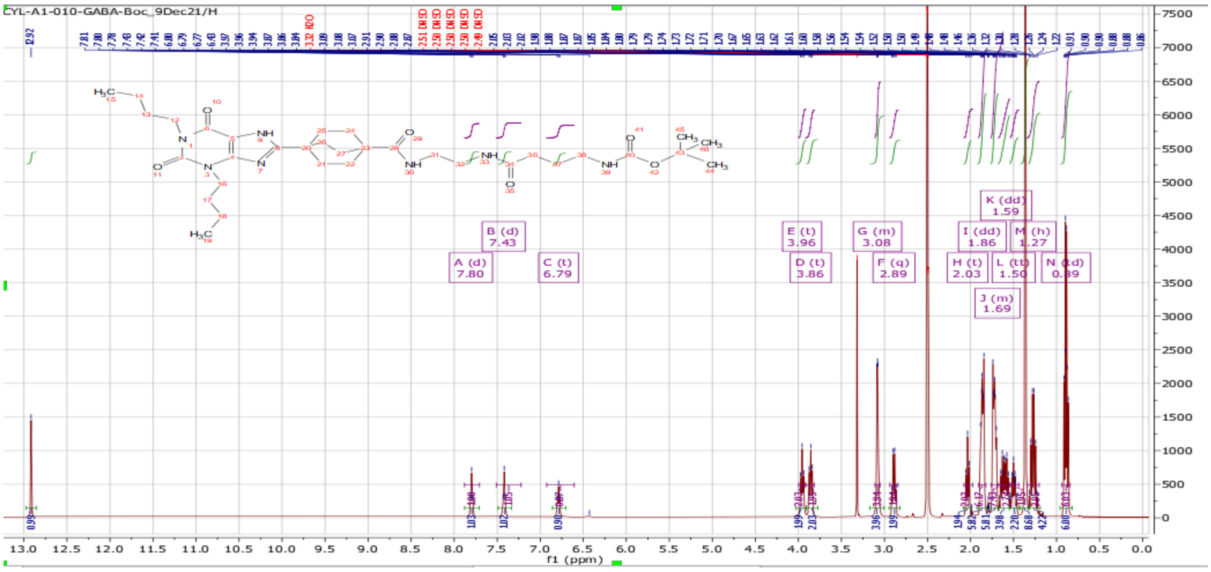

^{13}C NMR (101.62 MHz, CD$_3$OD) of **1**

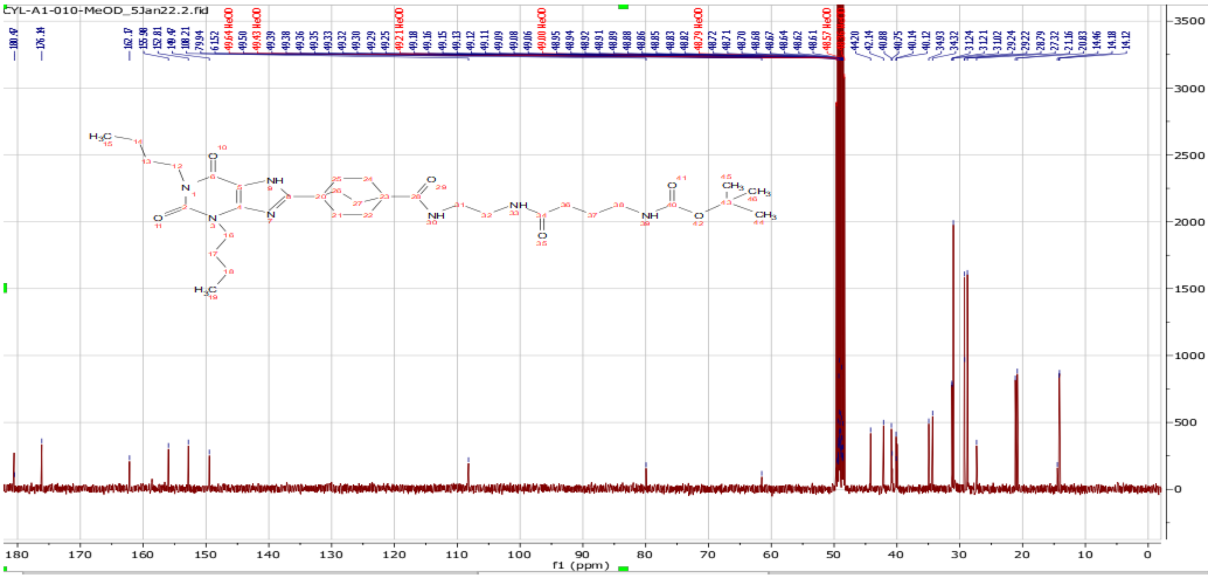

^{1}H NMR (400 MHz, CD_3OD) of **3**

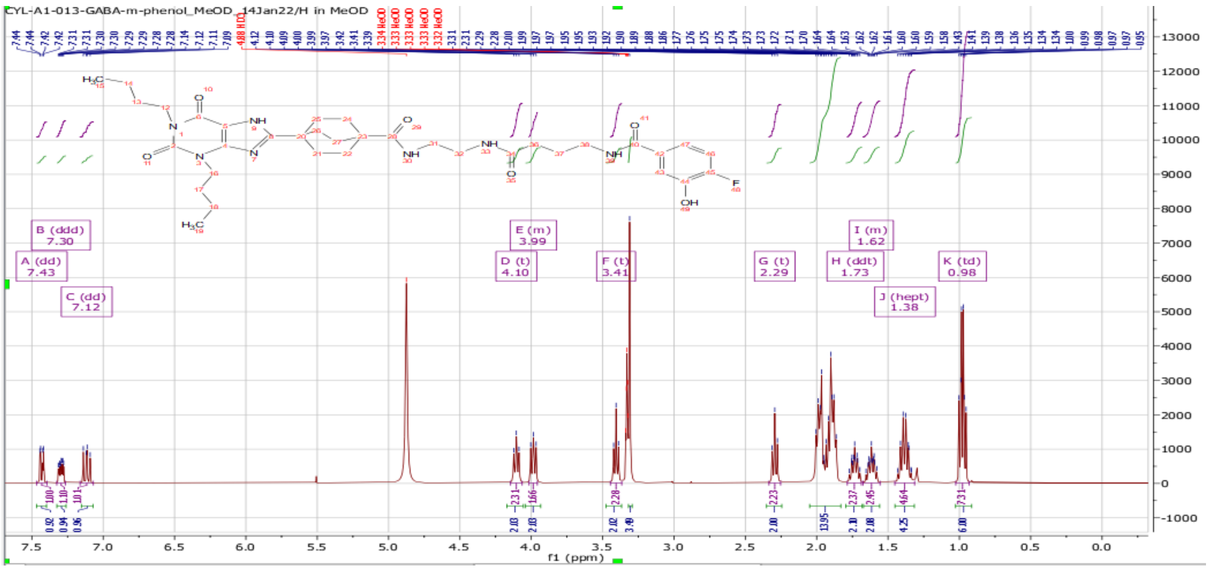

^{13}C NMR (101.62 MHz, CD_3OD) of **3**

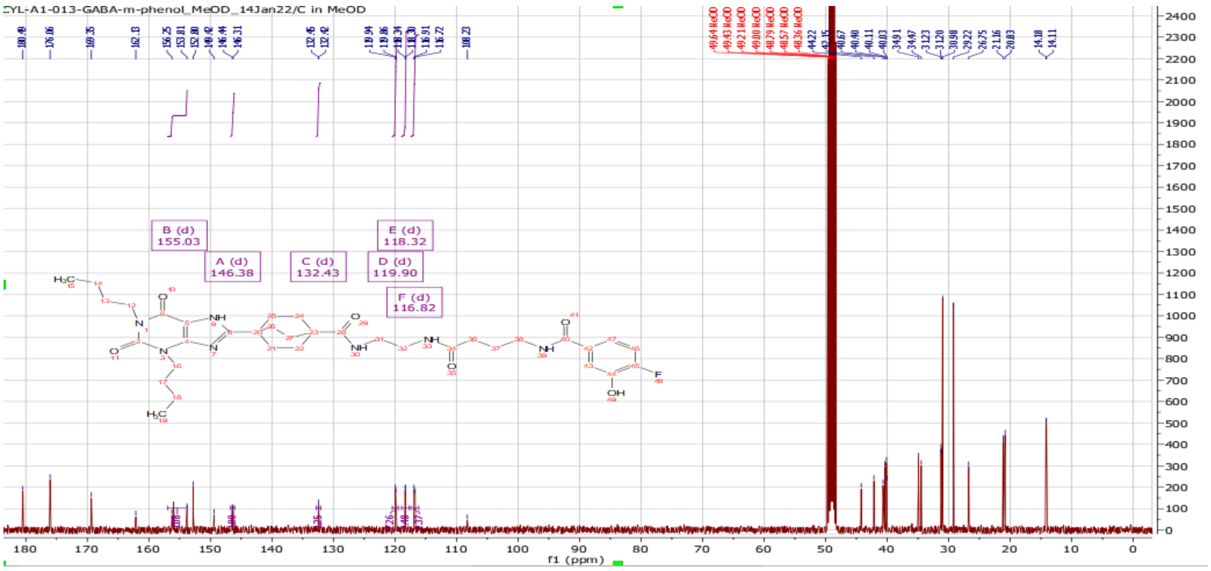

^{1}H NMR (400 MHz, $CDCl_3$) of **4**

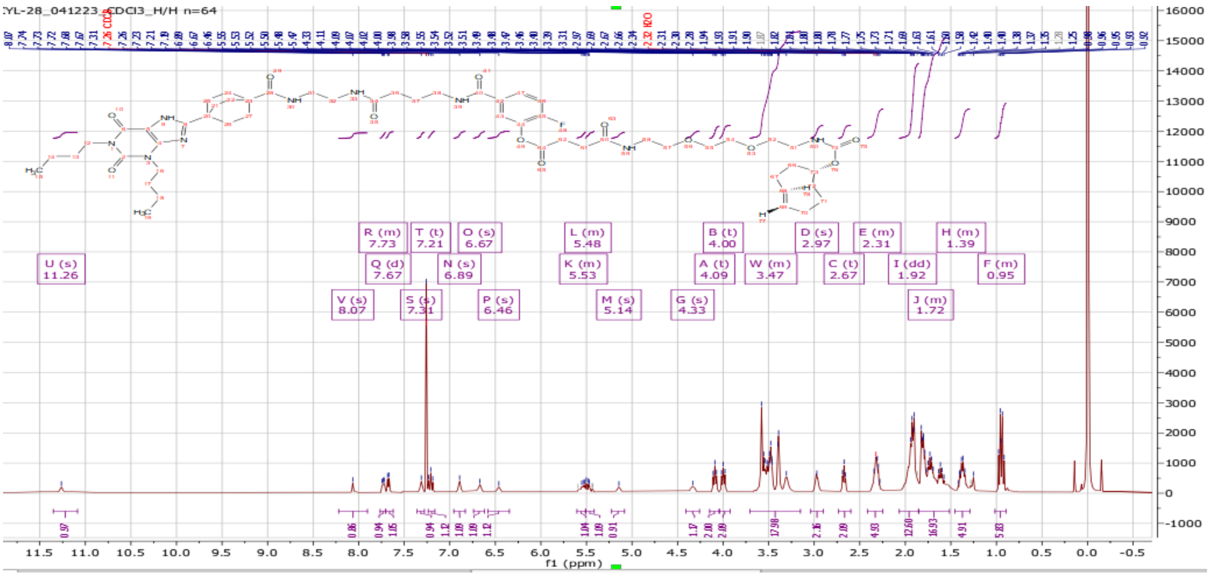

^{13}C NMR (101.62 MHz, $CDCl_3$) of **4**

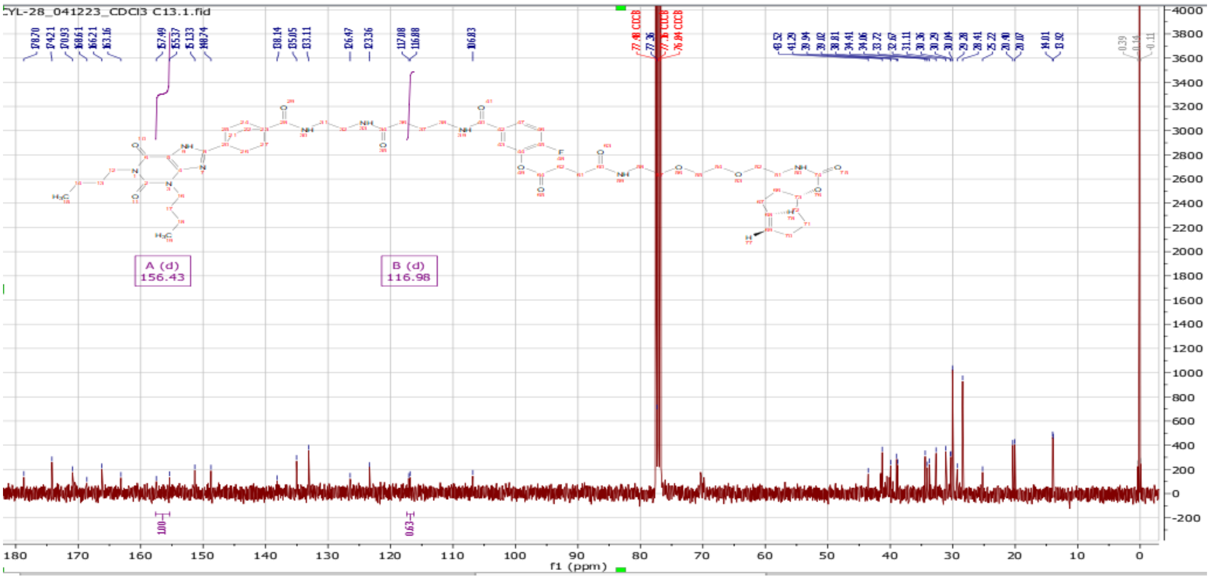

^{1}H NMR (400 MHz, CD_3OD) of **S6**

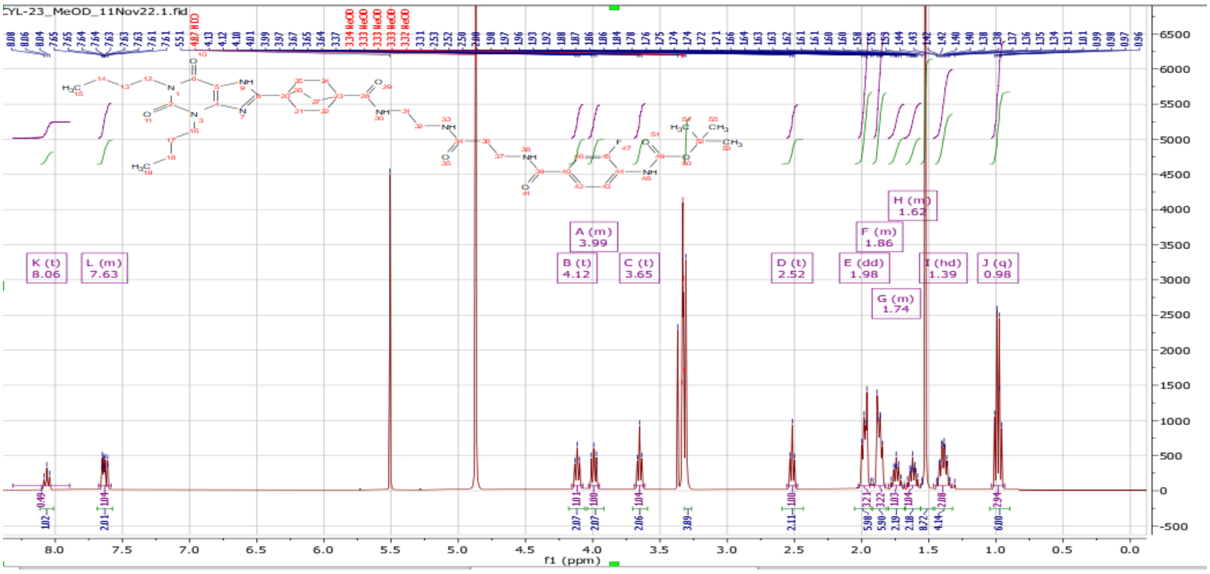

^{13}CNMR (101.62 MHz, $CDCl_3$) of **S6**

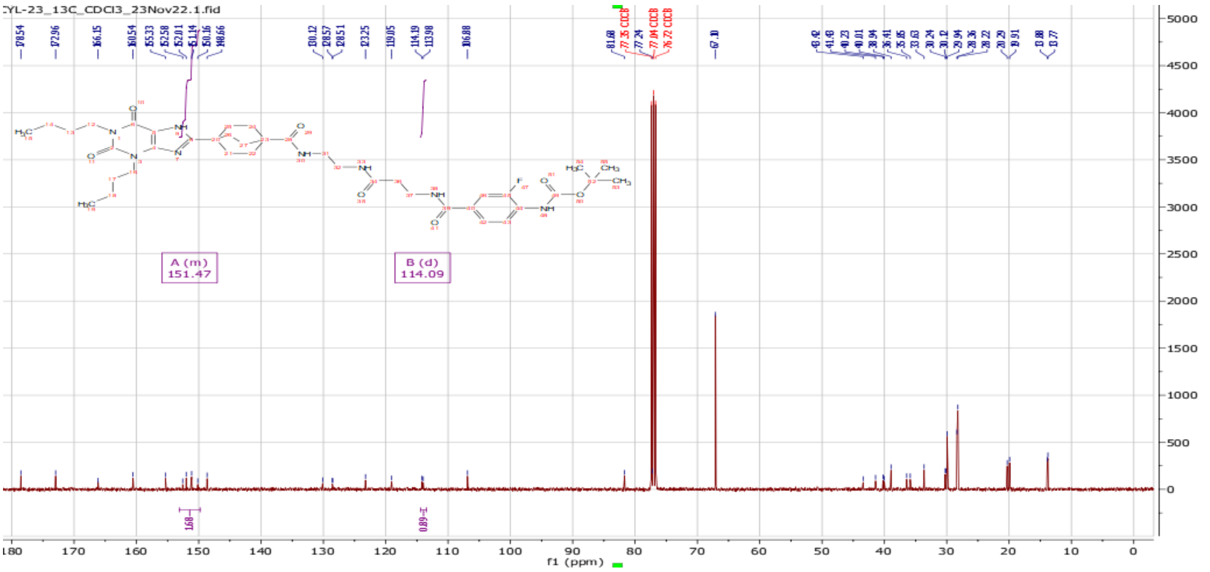

^{1}H NMR (400 MHz, CD_3OD) of **S8**

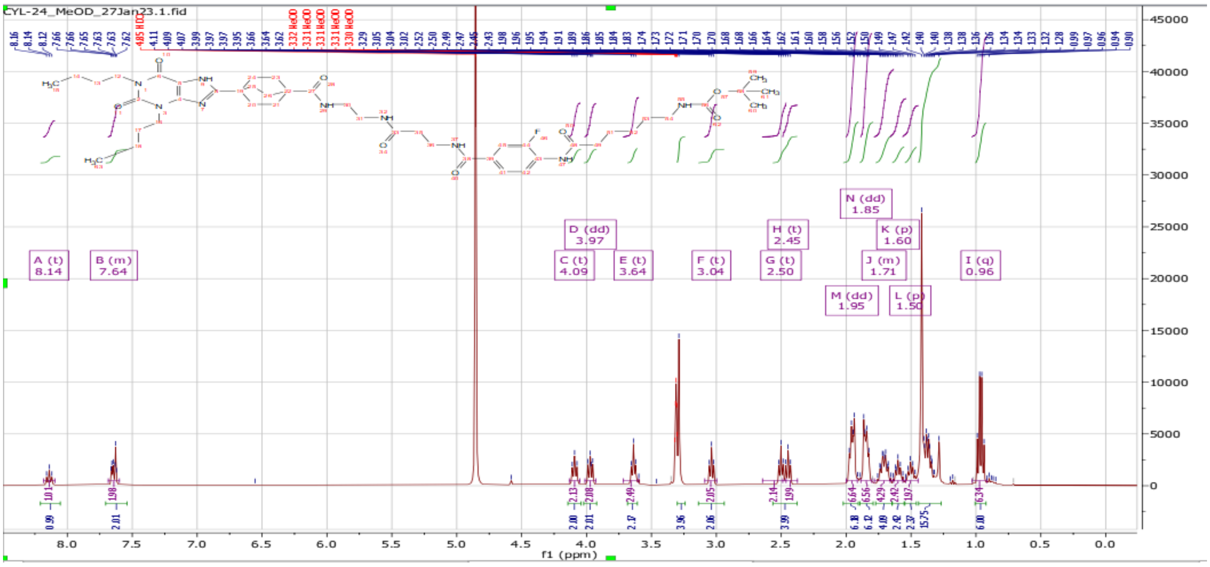

^{13}CNMR (101.62 MHz, CD_3OD) of **S8**

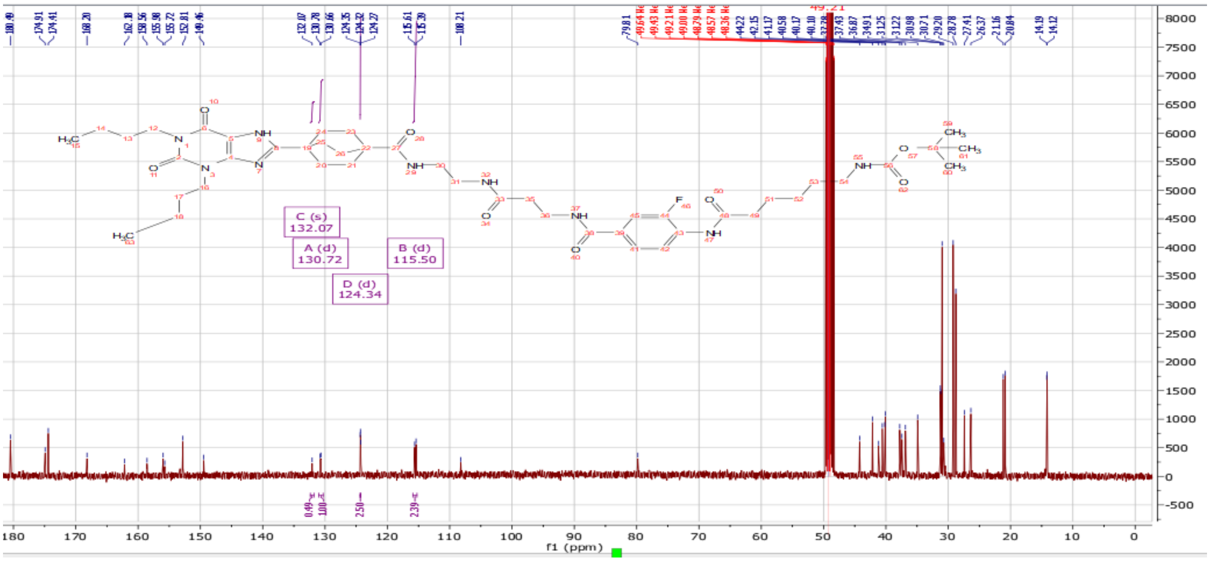

^{1}H NMR (400 MHz, DMSO-d_6) of **S11**

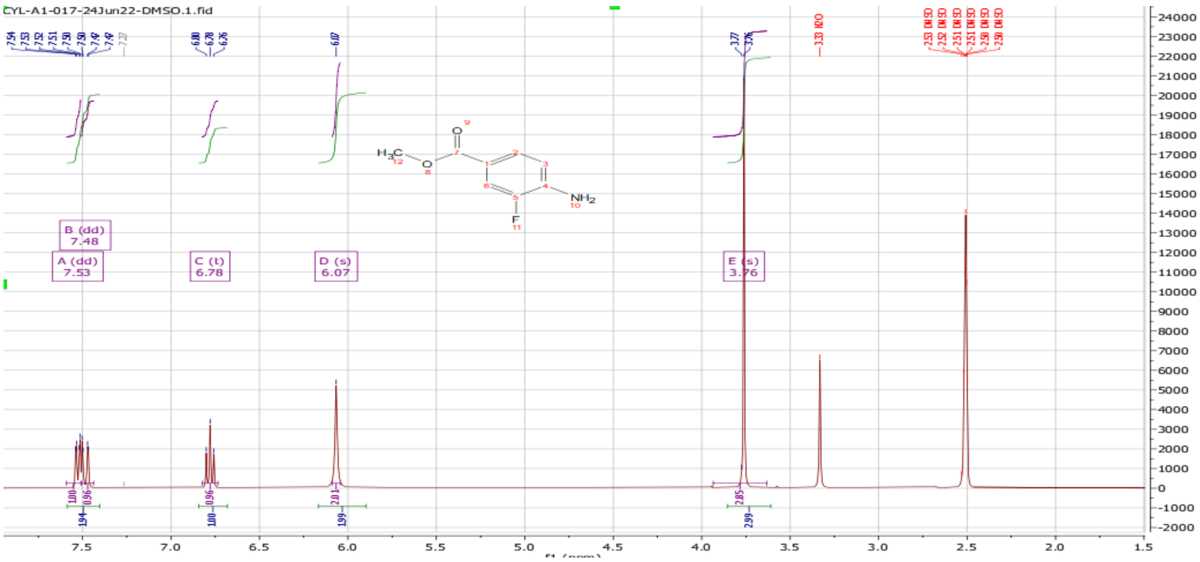

^{13}CNMR (101.62 MHz, DMSO-d_6) of **S11**

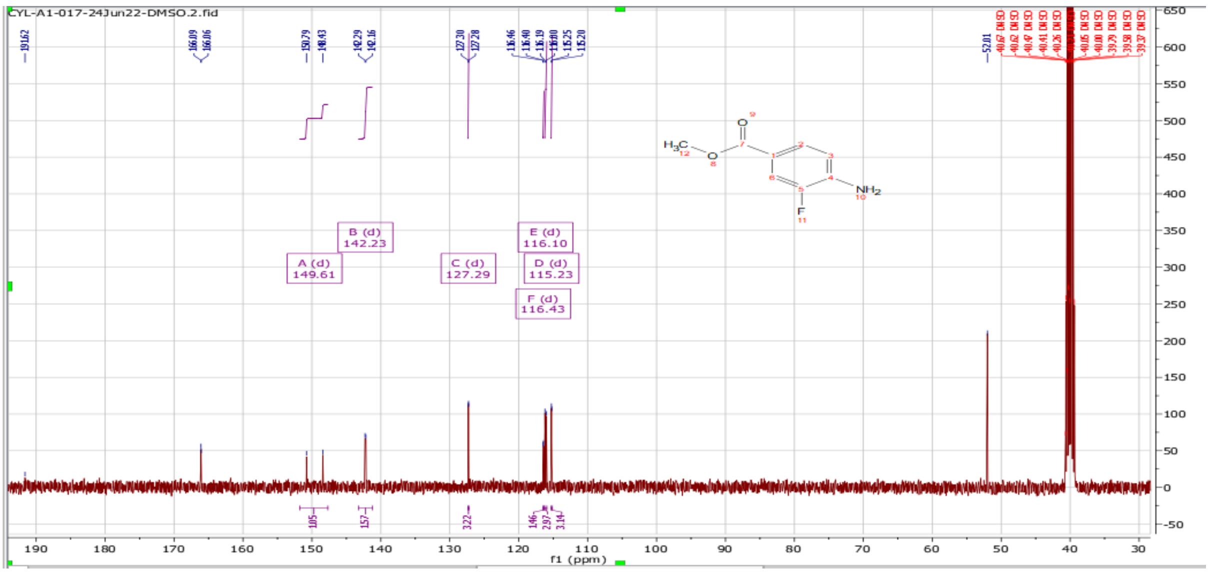

^{1}H NMR (400 MHz, DMSO-d_6) of **S12**

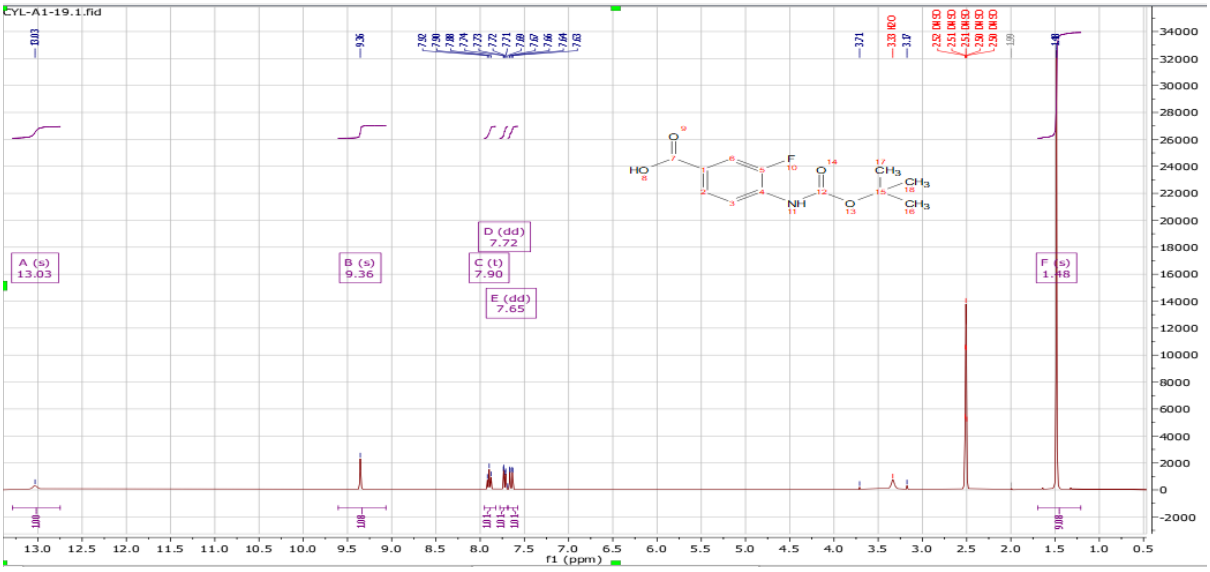

^{13}CNMR (101.62 MHz, DMSO-d_6) of **S12**

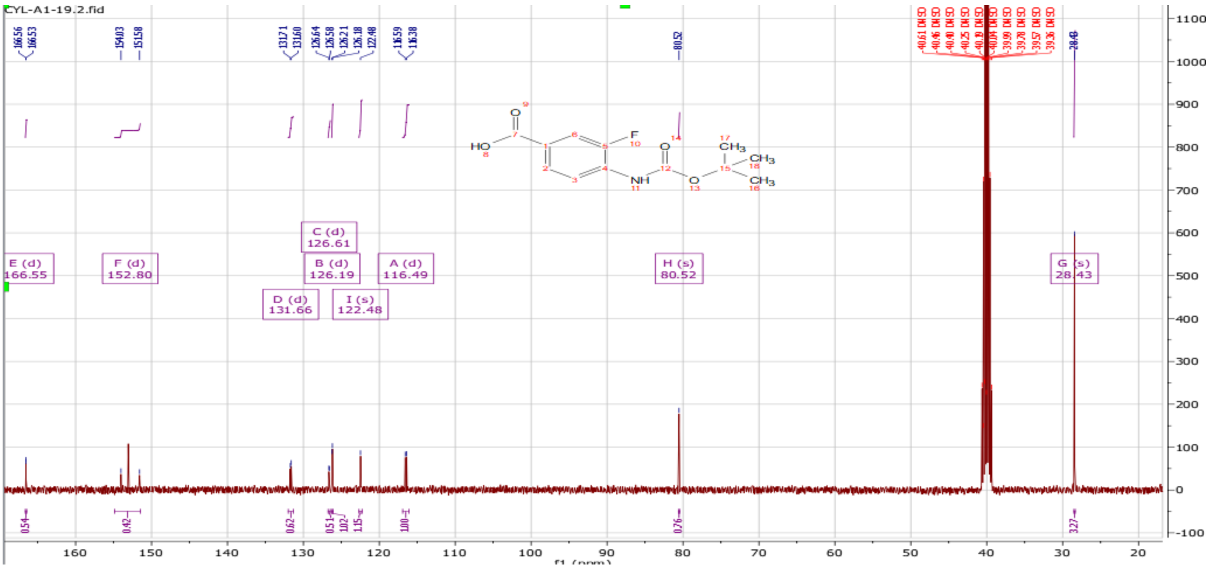

^{1}H NMR (400 MHz, $CDCl_3$) of **S14**

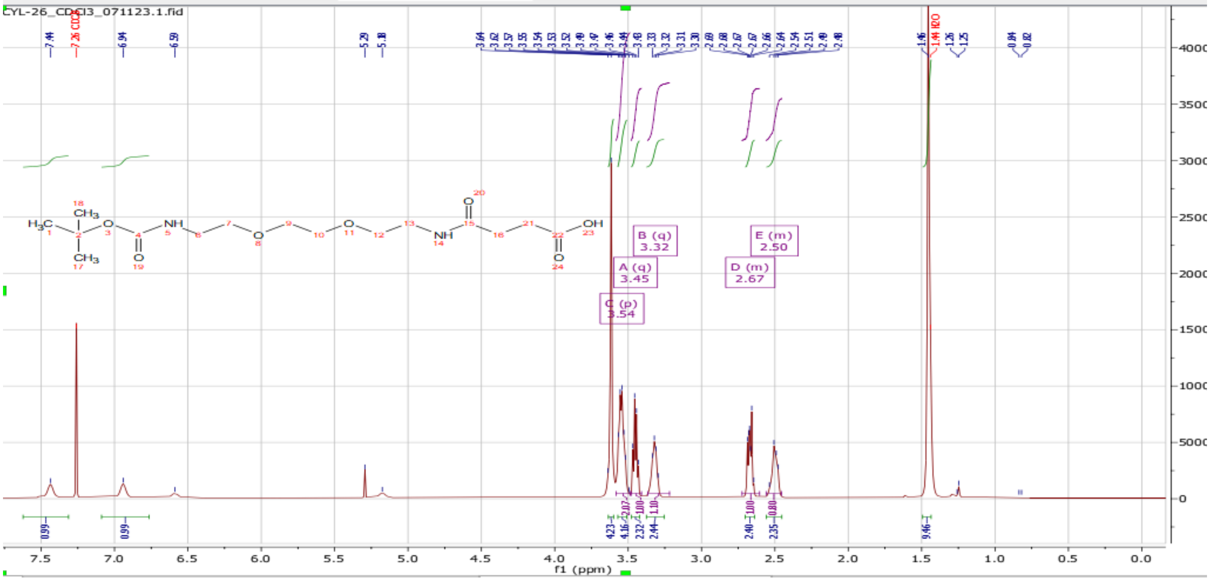

^{13}CNMR (101.62 MHz, $CDCl_3$) of **S14**

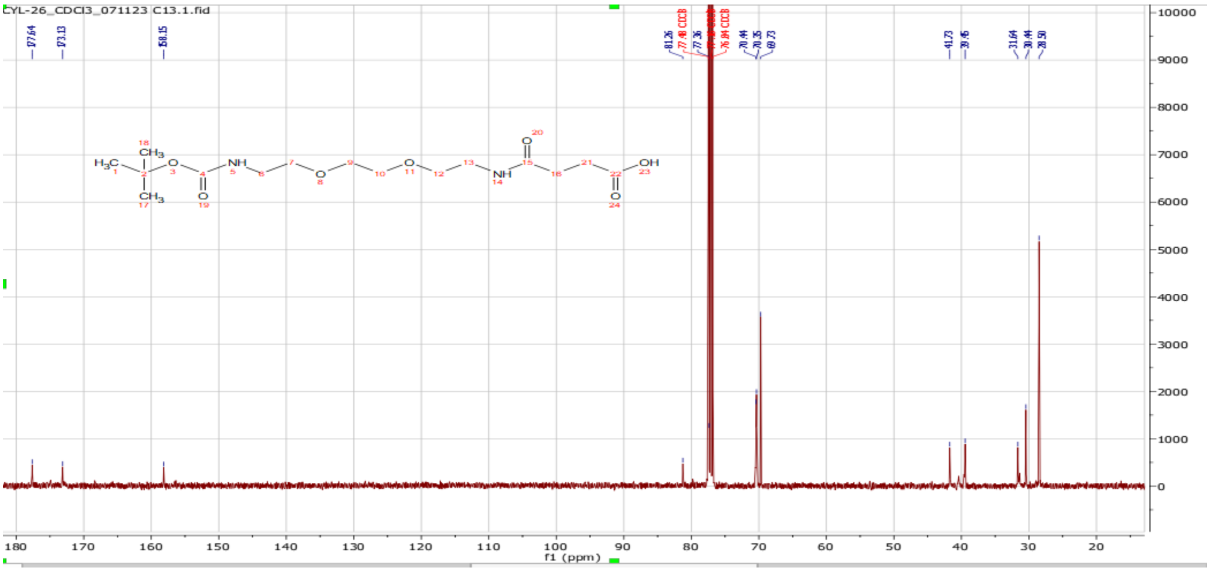

^{1}H NMR (400 MHz, CD_3OD) of **S15**

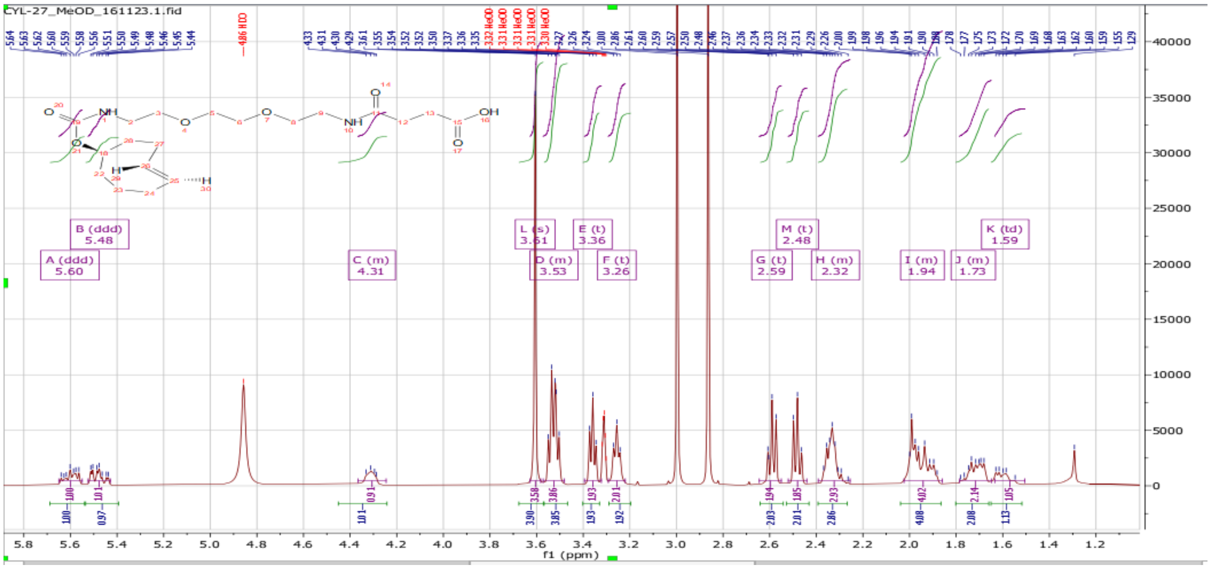

^{13}CNMR (101.62 MHz, CD_3OD) of **S15**

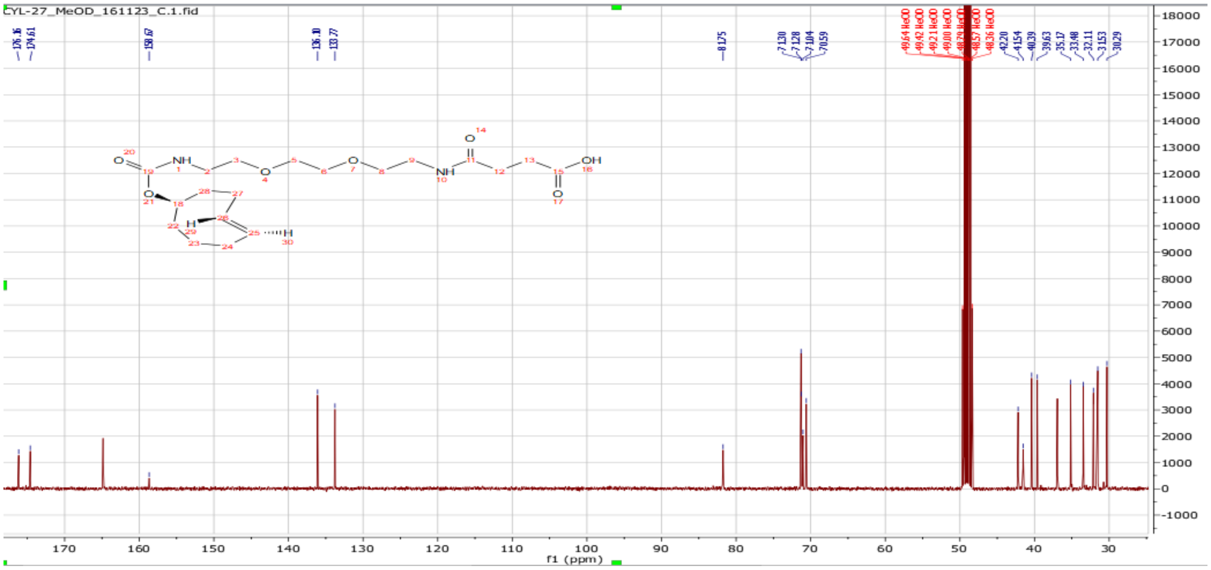

Supplement: Supplementary file 1 [file jm5c02389_si_001.pdf]
